# Effectiveness and safety of ranibizumab 0.5 mg in treatment-naïve patients with diabetic macular edema: Results from the real-world global LUMINOUS study

**DOI:** 10.1371/journal.pone.0233595

**Published:** 2020-06-03

**Authors:** Paul Mitchell, Tom G. Sheidow, Michel E. Farah, Sajjad Mahmood, Angelo M. Minnella, Nicole Eter, Bora Eldem, Hassan Al-Dhibi, Wayne Macfadden, Soumil Parikh, Cornelia Dunger-Baldauf, Mohamed M. Mahgoub, Ursula Schmidt-Erfurth, on behalf of the LUMINOUS study investigators

**Affiliations:** 1 Centre for Vision Research, Department of Ophthalmology and Westmead Institute for Medical Research, University of Sydney, Sydney, NSW, Australia; 2 Ivey Eye Institute, Schulich School of Medicine, Western University, Ontario, Canada; 3 Department of Ophthalmology, Federal University of São Paulo, Paulista School of Medicine, São Paulo, Brazil; 4 Royal Eye Hospital, Oxford Road, Manchester, United Kingdom; 5 Institute of Ophthalmology, Catholic University "Sacro Cuore" Foundation "A. Gemelli", Rome, Italy; 6 Department of Ophthalmology, University of Muenster Medical Center, Muenster, Germany; 7 Department of Ophthalmology, Hacettepe University, Faculty of Medicine, Sıhhiye, Ankara, Turkey; 8 Vitreoretinal and Uveitis Division, King Khaled Eye Specialist Hospital, Riyadh, Kingdom of Saudi Arabia; 9 Novartis Pharma AG, Basel, Switzerland; 10 Ain-Shams University, Universidad de El Cairo, Cairo, Egypt; 11 Department of Ophthalmology and Optometry, Medical University of Vienna, Vienna, Austria; University of Florida, UNITED STATES

## Abstract

**Purpose:**

To assess the one-year effectiveness and safety of ranibizumab 0.5 mg in treatment- naïve patients with diabetic macular edema (DME) enrolled in the real-world LUMINOUS study.

**Patients and methods:**

A 5-year, prospective, observational, open-label, global study which recruited 30,138 patients across all approved indications. Consenting patients (≥18 years) who were treatment-naïve or previously treated with ranibizumab or other ocular treatments were treated as per the local ranibizumab label. Here, we present the change in visual acuity (VA) (Early Treatment Diabetic Retinopathy Study letter score; primary treated eye) at Year 1, as well as the change in VA based on injection frequencies (≤4 and ≥5), treatment exposure, and the overall adverse events (AEs) and serious AEs (SAEs) in treatment-naïve DME patients.

**Results:**

Of the 4,710 DME patients enrolled in the study, 1,063 were treatment-naïve. At baseline, mean age was 64.5 years, 54.7% were male, and 69.2% were white. At 1 year, mean VA letter score improved by +3.5 (n = 502) from a baseline of 57.7 with a mean of 4.5 injections. Presented by injection frequencies ≤4 and ≥5, VA letter score gains were 0.5 (n = 264) and 6.9 (n = 238) from baseline letter scores of 56.6 and 59.0, respectively. Over 5 years, the incidence of ocular/non-ocular AEs and SAEs was 7.2%/10.1% and 0.3%/5.8%, respectively. No endophthalmitis cases were reported.

**Conclusions:**

The LUMINOUS study included patients with DME with more diverse baseline characteristics than those in randomized clinical trials. The 1-year data showed improvement in VA with low number of injections in treatment- naïve patients with DME. Greater VA gains were observed in patients who received ≥5 injections. No new safety findings were identified. LUMINOUS confirms the effectiveness and safety of ranibizumab for the treatment of patients with DME in a real-world clinical practice.

## Introduction

Diabetic macular edema (DME), a common manifestation of diabetic retinopathy (DR), is a leading cause of vision impairment in diabetic patients if left untreated [1, 2]. The burden of DME is likely to increase in future as the diabetic population is projected to rise worldwide from a current estimate of 415 million to an estimate of 642 million by 2040 [3–6].

The introduction of anti-vascular endothelial growth factor (VEGF) agents such as ranibizumab has revolutionized the treatment of patients with visual impairment due to DME [7] and several randomized controlled trials (RCTs) have established the efficacy and safety of ranibizumab 0.5 mg in this patient population [8–11]. However, the findings from RCTs do not always correlate with that of routine clinical practice due to differences from RCTs, which typically include a smaller and more homogeneous patient population, prescribed treatment patterns, and shorter observational periods [12, 13]. Real-world evidence (RWE) in more heterogeneous populations therefore complements evidence from RCTs to better inform physicians regarding treatment practices and patterns in routine clinical settings [14].

The LUMINOUS™ (NCT01318941) study (study investigators are listed in S1 Appendix), the largest prospective, observational study in medical retina, was designed to evaluate the long-term effectiveness, safety, and treatment patterns with ranibizumab 0.5 mg in routine clinical practice across all approved indications [15]. Here, we present global and country-specific results for the treatment-naïve subgroup of patients with DME enrolled in LUMINOUS.

## Materials and methods

### Study design

LUMINOUS was a 5-year, prospective, observational, multicenter, open-label, single-arm, global study and conducted from March 2011 to April 2016 at 488 centers across 42 countries [15]. In the protocol, the time period between study start and completion was pre-defined as 5 years.

Patients with any of the indications as per the approved ranibizumab label were enrolled. These patients were treated with intravitreal ranibizumab 0.5 mg according to the local product label at outpatient public or private ophthalmology clinics. As patients were recruited over time and the calendar time point of study completion was pre-set, follow-up time varied according to the entry dates. The minimum potential follow-up for each patient was defined as one year in the protocol. Visits took place at a frequency as determined by the investigator. Data from all visits were documented in electronic case report format (eCRF). However, it was recommended that data should be captured in the eCRF at every visit or a minimum of every 3 months and physicians were encouraged to follow-up with patients who had not been seen in the clinic for at least 6 months since the previous visit, in order to capture data. Patients not seen at least once per year, or those switched to another anti-VEGF therapy, were discontinued from the study.

The first eye treated during the study was considered the primary treated eye. If both eyes were first treated on the same date, or if both eyes were pre-treated, the eye with the earliest diagnosis date was considered the primary treated eye. If both eyes had the same diagnosis date, one eye was chosen randomly as the primary treated eye.

The study protocol and the proposed informed consent form were reviewed and approved by an Independent Ethics Committee and/or Institutional Review Board for each center. The details of Independent Ethics Committee and/or Institutional Review Board are provided in S2 Appendix. The study was designed, implemented, and reported in accordance with the Guidelines for Good Pharmacoepidemiology Practices issued by the International Society for Pharmacoepidemiology, with any applicable national guidelines, and ethical principles laid down in the Declaration of Helsinki. Patients provided written informed consent. The study is registered with Clinicaltrials.gov (identifier, NCT01318941) [15].

### Study population

Consenting adult (≥18 years old) patients who were either treatment-naïve, or previously treated with ranibizumab, or another ocular therapy for any of the approved indications included in the local product label, were enrolled. Patients were excluded if they were participating in other investigational studies or if they had received systemic or ocular anti-VEGF therapy other than ranibizumab 90 days or 30 days prior to enrollment, respectively. Further details of the inclusion and exclusion criteria have been reported elsewhere.

### Assessments

Effectiveness assessments included VA (preferably best-corrected VA [BCVA]) evaluation by each participating physician as a part of routine care practice using Early Treatment Diabetic Retinopathy Study (ETDRS) letters or Snellen charts. To facilitate data analysis, Snellen fractions and decimals were converted to ETDRS equivalent letter scores. It was recommended that the same method of VA assessment be used throughout the study wherever possible. All AEs, including serious AEs (SAEs), irrespective of suspected causal association that occurred during the study, were also collected.

Other assessments such as optical coherence tomography (to measure central retinal thickness) and ocular examination (for pre-injection intraocular pressure) were optional, but were included when the data were available.

The number of ranibizumab injections administered overall, over time, and the average time interval (in weeks) between consecutive injections, visit frequency, treatment patterns, unilateral (involving single eye)/bilateral (involving both eyes) treatments, and proportion of patients receiving both ocular and non-ocular concomitant medications were recorded.

### Statistical analysis

Due to the design of the study, 1 year data were available potentially for all patients, while the availability of data for subsequent years depended on the patient’s study entry date. The effectiveness data are therefore presented here for the time period up to 1 year. Effectiveness data following more than 1 year of ranibizumab treatment in patients with DME will be described separately.

All effectiveness and safety data were summarized descriptively. The enrolled set included all patients who signed the informed consent, and had at least the baseline assessment. The safety set comprised patients in the enrolled set who were treated with at least one dose of ranibizumab during the study or prior to the start of the study and had at least one safety assessment after the first treatment. The primary treated eye set included all primary treated eyes of patients from the safety set and was the primary analysis set for effectiveness.

For treatment-naïve eyes, the date of first on-study injection with ranibizumab was considered the baseline date (study day 1). The primary effectiveness variable was the mean change in VA (ETDRS letter score) from baseline presented by quarterly and yearly periods for the primary treated eye set. Effectiveness data are presented only for patients in the primary treated eye set for whom baseline and year 1 data were captured. The mean change in visual acuity (VA) from baseline at year 1 was presented by baseline injection frequency (≤4 and ≥5), use of loading (at least three ranibizumab injections up to day 100) and non-loading doses, and baseline VA category. Further VA evaluations included the proportion of patients maintaining baseline VA of ≥73 letters (good starting vision or Snellen equivalent 20/40) at year 1 and those achieving VA of ≥73 from a baseline VA of <73 letters (poorer starting vision) at year 1; the proportion of patients with a VA loss (defined as ≤0 letter change from baseline) or gain (defined as >0 letter change from baseline) of >0 to <5 letters, ≥5 to <10 letters, ≥10 to <15 letters, and ≥15 letters at year 1. The number of injections and monitoring visits up to 1 year were summarized for patients who had participated in the study for at least 365 days. Safety was assessed based on the incidence proportion, relationship, and severity of treatment-emergent ocular and non-ocular AEs during the defined time periods. Ocular AEs were assessed for the primary treated eye set and non-ocular AEs were assessed for the safety set over 5 years.

## Results

The number of patients recruited and treated with ranibizumab (safety set) in the LUMINOUS study was 30,138 patients across all the approved indications worldwide. Of these, 4,710 patients had DME: 1,063 were treatment-naïve, 2,519 were treatment non-naïve (already under ranibizumab treatment), and 1,128 were treatment non-naïve (other ocular treatments). Of the 1,063 treatment-naïve patients, 893 (85.1%) remained in the study at 1 year; the main reasons for discontinuation were patient loss to follow-up (7.0%), switch to anti-VEGF other than ranibizumab (1.8%), or DME no longer required study drug (1.5%; Table 1). Not all patients remaining in the study at year 1 had a 1 year VA value recorded (a value recorded within a 1.5 month window around 12 months); as per design of the study, visits were scheduled at the discretion of the investigator and could fall outside the 12 months window. The primary treated eye set included 502 patients with both baseline and 1-year VA data. The safety set comprised 1,063 treatment-naïve DME patients.

**Table 1:** Patient disposition of treatment-naïve patients with DME (safety set).

Disposition, n (%)	Treatment-naïve DME patients (N = 1,049*)
Patients with one eye treated during the study	677 (64.5)
Patient ongoing in the study at year 1	893 (85.1)
Patients who discontinued the study	156 (14.9)
**Reasons for discontinuation**	
AEs	4 (0.4)
Abnormal laboratory values	0
Abnormal test procedure results	0
Unsatisfactory therapeutic effect	13 (1.2)
Subject’s condition no longer required study drug	16 (1.5)
Subject withdrew consent	12 (1.1)
Lost to follow-up	73 (7.0)
Administrative problems	5 (0.5)
Death	12 (1.1)
Pregnancy	0
Subject switched to anti-VEGF other than ranibizumab	19 (1.8)
Protocol deviation	2 (0.2)

The safety set consisted of patients in the enrolled set who were treated with at least 1 dose of ranibizumab during this study or prior to start of study and had at least 1 safety assessment after the first treatment.

*Patients with a baseline visit on or before 1 March, 2015 are included. Data missing for 14 patients from the total 1,063 enrolled treatment-naïve patients with DME.

Data collected until the last recorded follow-up date was used to perform the analyses.

For treatment-naïve eyes, the date of first on-study injection with ranibizumab was considered the baseline date.

AE, adverse event; DME, diabetic macular edema; VEGF, vascular endothelial growth factor

At baseline, the mean (standard deviation [SD]) age of the treatment-naïve DME patients (N = 1,063) was 64.5 (11.1) years, most were male (54.7%) and 69.2% were white (Table 2). Relevant ocular medical history and current medical conditions were reported for 27.6% of treatment-naïve eyes (Table 3). Concomitant ocular medications and significant non-drug therapies were reported in 19.4% of patients in the primary treated eye set whereas concomitant non-ocular medications and significant non-drug therapies were reported in 79.5% of patients in the safety set. In the safety set, 84.8% of treatment-naïve patients had relevant non-ocular medical history and current medical conditions (Table 3). The mean (SD) glycated hemoglobin (HbA_1c_) at baseline was 7.9% (3.4) (Table 3). All patients with DME have, or have had diabetes and/or diabetic retinopathy. However, many Investigators did not identify diabetes as comorbid conditions, per their discretion. The normal range of HbA_1c_ is usually from 4.5%–6.2% [16, 17] and the baseline value of these treatment-naïve patients with DME indicates that the diabetes mellitus of these patients was under reasonable to fair control, on average.

**Table 2:** Baseline demographic, ocular, and disease characteristics for treatment-naïve DME patients (primary treated eye set).

Variables/characteristics	Treatment-naïve DME patients (N = 1,063)
Mean (SD) age, years	64.5 (11.1)
Gender, Male, n (%)	581 (54.7)
**Race, n (%)**	
White	736 (69.2)
Black	9 (0.8)
Asian	133 (12.5)
Native American	38 (3.6)
Pacific Islander	2 (0.2)
Other	105 (9.9)
Missing	40 (3.8)
Median time from diagnosis, days	30.0
VA, letters, n	926
Mean (SD)	56.3 (18.7)
**VA (letters) categories, n (%)**	
<23	59 (5.6)
23 –<39	126 (11.9)
39 –<60	244 (23.0)
60 –<74	323 (30.4)
≥74	174 (16.4)
Missing	137 (12.9)
Central retinal thickness,* μm, n	739
Mean (SD)	420 (135.8)

The primary treated eye set included all primary treated eyes in patients included in the safety set. Patients with a baseline visit date are included.

The safety set consisted of patients in the enrolled set who were treated with at least one dose of ranibizumab during this study or prior to the start of the study and had at least one safety assessment after the first treatment.

*Assessed by time domain and spectral domain optical coherence tomography.

Data collected until the last recorded follow-up date was used to perform the analyses.

For treatment-naïve eyes, the date of first on-study injection with ranibizumab was considered the baseline date.

DME, diabetic macular edema; SD, standard deviation; VA, visual acuity.

**Table 3:** Relevant ocular and non-ocular medical history/comorbidities for treatment-naïve DME patients.

Variables/characteristics	Treatment-naïve DME patients (N = 1,063)
**Ocular medical history/comorbidities, total, n (%)**	293 (27.6)
Diabetic retinopathy	133 (12.5)
Diabetic retinal edema	59 (5.6)
Macular edema	49 (4.6)
Cataract	79 (7.4)
Cataract operation	47 (4.4)
**Non-ocular medical history/comorbidities, total, n (%)**	901 (84.8)
Diabetes mellitus	659 (62.0)
Hypercholesterolemia	197 (18.5)
Type 2 diabetes mellitus	147 (13.8)
Obesity	77 (7.2)
Hyperlipidemia	57 (5.4)
Hypertension	512 (48.2)
Myocardial infarction	76 (7.2)
Stroke	52 (4.9)
HbA_1c_, %	
n	450
Mean (SD)	7.9 (3.4)

Primary treated eye set for ocular medical history/comorbidities.

Safety set for non-ocular medical history/comorbidities.

The primary treated eye set included all primary treated eyes in patients included in the safety set. Patients with a baseline visit date are included.

The safety set consisted of patients in the enrolled set who were treated with at least one dose of ranibizumab during this study or prior to the start of the study and had at least one safety assessment after the first treatment.

Patients with a baseline visit date are included.

Patient numbers ≥4% are included in this table.

Data collected until the last recorded follow-up date was used to perform the analyses.

For treatment-naïve eyes, the date of first on-study injection with ranibizumab was considered the baseline date.

DME, diabetic macular edema; SD, standard deviation; VA, visual acuity.

**Fig 1:**
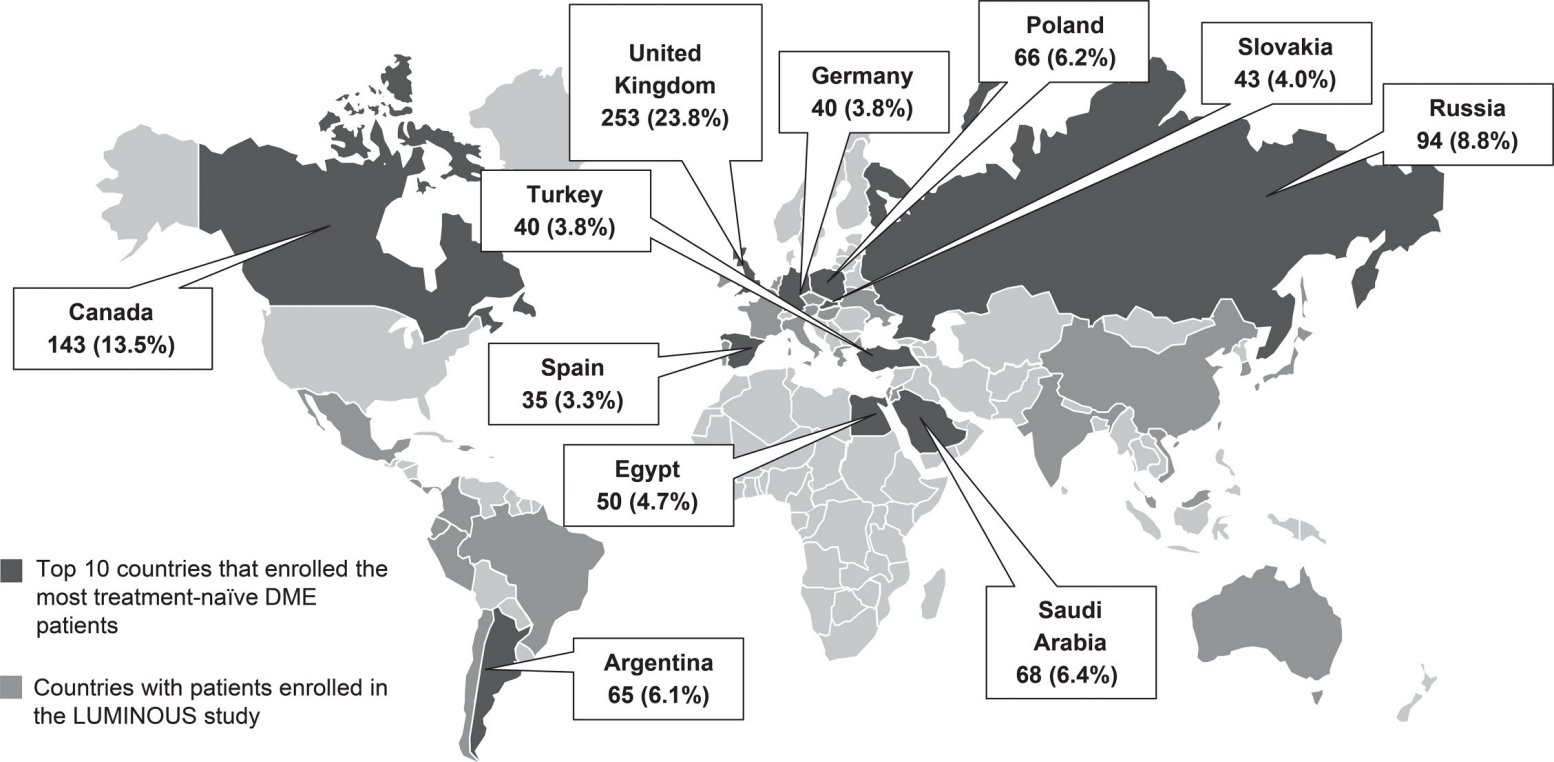
LUMINOUS study overview: World map showing overall recruitment with countries enrolling highest treatment-naïve patients with DME. The pop out boxes represent the number (%) of treatment-naïve patients with DME in the top. Equal recruitment numbers were achieved in Turkey and Germany. DME, diabetic macular edema.

The countries that enrolled the most treatment-naïve patients with DME were the United Kingdom (UK; 23.8%), Canada (13.5%), Russia (8.8%), Saudi Arabia (6.4%), Poland (6.2%), Argentina (6.1%), Egypt (4.7%), Slovakia (4.0), Germany and Turkey (each 3.8%), and Spain (3.3%; Fig 1). The baseline demographics, ocular, and disease characteristics of treatment-naïve patients with DME from these countries were similar to the overall characteristics.

### Efficacy outcomes

**Fig 2:**
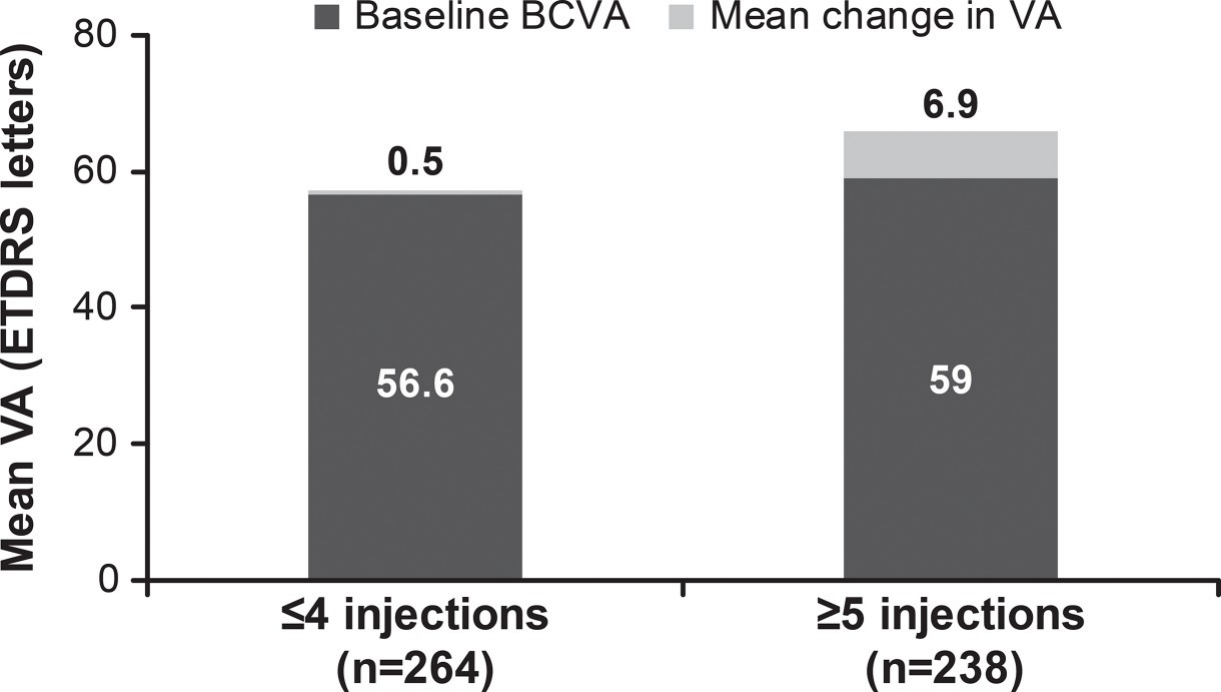
Mean change in visual acuity by injection frequency in treatment-naïve patients with DME (N = 502; primary treated eye set). Observed dataset for VA change. The primary treated eye set included all primary treated eyes in patients included in the safety set. The safety set consisted of patients in the enrolled set who were treated with at least one dose of ranibizumab during this study or prior to the start of the study and had at least one safety assessment after the first treatment. For treatment-naïve eyes, the date of first on-study injection with ranibizumab was considered the baseline date. For the 1-year time period, all patients with non-missing baseline VA and year 1 VA performed anywhere between Day 319 and Day 409 but who had been in the study for at least 365 days from baseline to last follow-up date were included in the analysis. DME, diabetic macular edema; ETDRS, Early Treatment Diabetic Retinopathy Study; VA, visual acuity.

**Fig 3:**
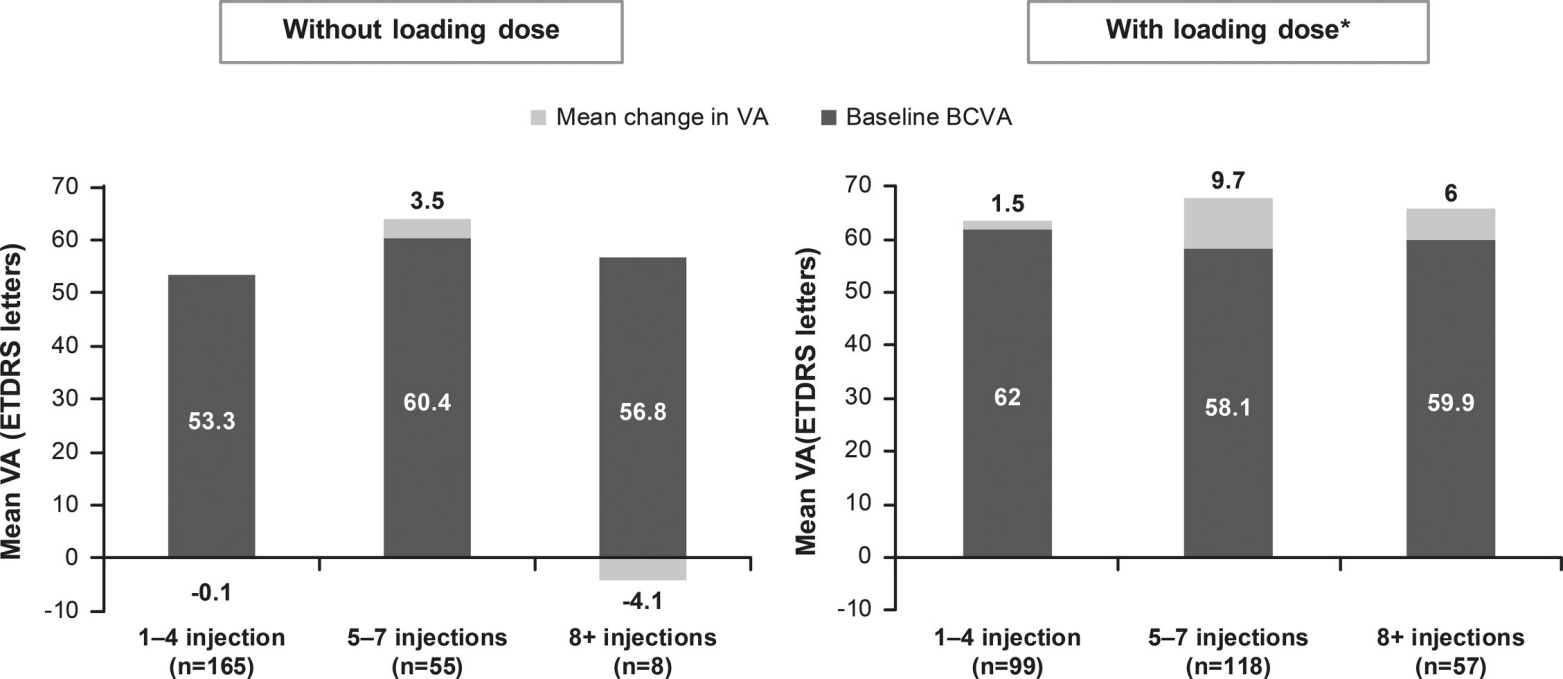
Mean change in visual acuity in patients with and without loading doses (primary treated eye set). *Three initial consecutive injections at 4 weekly intervals. Observed dataset for VA change. The primary treated eye set included all primary treated eyes in patients included in the safety set. The safety set consisted of patients in the enrolled set who were treated with at least one dose of ranibizumab during this study or prior to the start of the study and had at least one safety assessment after the first treatment. For treatment-naïve eyes, the date of first on-study injection with ranibizumab was considered the baseline date. For the 1-year time period, all patients with non-missing baseline VA and year 1 VA performed anywhere between Day 319 and Day 409 but who had been in the study for at least 365 days from baseline to last follow-up date were included in the analysis.BCVA, best-corrected visual acuity; ETDRS, Early Treatment Diabetic Retinopathy Study; VA, visual acuity.

**Fig 4:**
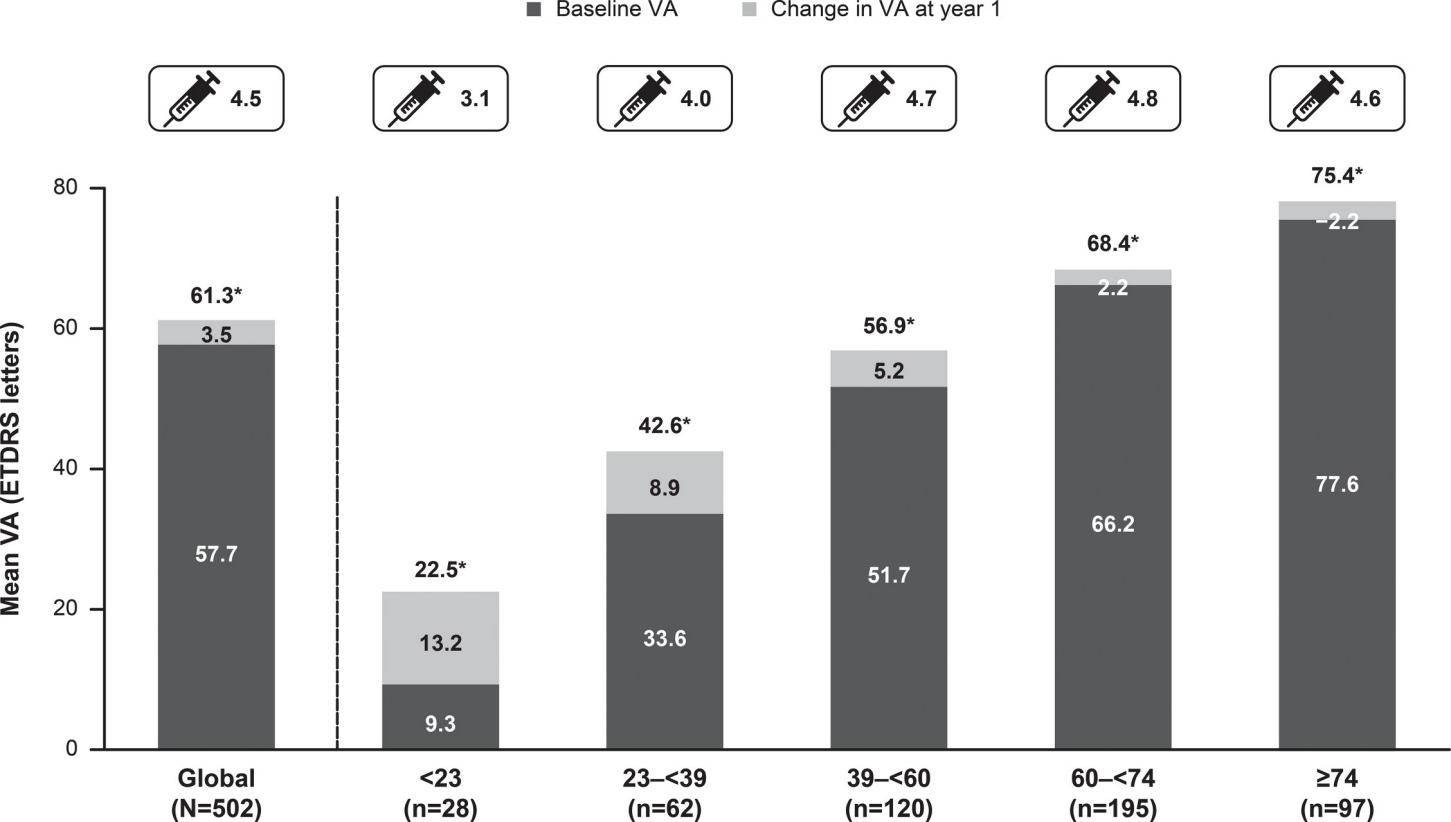
Mean change in visual acuity by baseline visual acuity stratification (primary treated eye set). Observed data set for VA change. The primary treated eye set included all primary treated eyes in patients included in the safety set. The safety set consisted of patients in the enrolled set who were treated with at least one dose of ranibizumab during this study or prior to the start of the study and had at least one safety assessment after the first treatment. *The values represent the final VA at year 1. For treatment-naïve eyes, the date of first on-study injection with ranibizumab was considered the baseline date. For the 1-year time period, all patients with non-missing baseline VA and year 1 VA performed anywhere between Day 319 and Day 409 but who had been in the study for at least 365 days from baseline to last follow-up date were included in the analysis. ETDRS, Early Treatment Diabetic Retinopathy Study; VA, visual acuity.

At year 1, there was a mean VA letter score gain of +3.5 from a baseline VA of 57.7 in treatment-naïve DME patients (n = 502). When presented by injection frequency, better VA letter score gains (+6.9) were observed in treatment-naïve DME patients receiving ≥5 injections (Fig 2). Patients who received loading dose, showed better VA gains compared with those who did not (Fig 3). Across all baseline VA categories, VA improved at year 1 except for those patients with baseline VA of ≥74 letters, in whom the VA was maintained (Fig 4). Although VA gains were greater in patients with lower baseline VA, the patients with higher baseline VA achieved the best final vision at 1 year (Fig 4).

There was a 14.8% increase in the proportion of treatment-naïve DME patients with VA ≥73 letters from baseline at month 12, regardless of their baseline VA. Of the 502 treatment-naïve DME patients completing one year, 99 (19.7%) had a baseline VA ≥73 letters and approximately 69% of these maintained this category of VA at 1 year. The other 403 (80.3%) patients of the 502 treatment-naïve DME patients had a baseline VA letter scores <73 and of these, 105 (26.1%) patients achieved VA letter scores ≥73 at Month 12.

**Fig 5:**
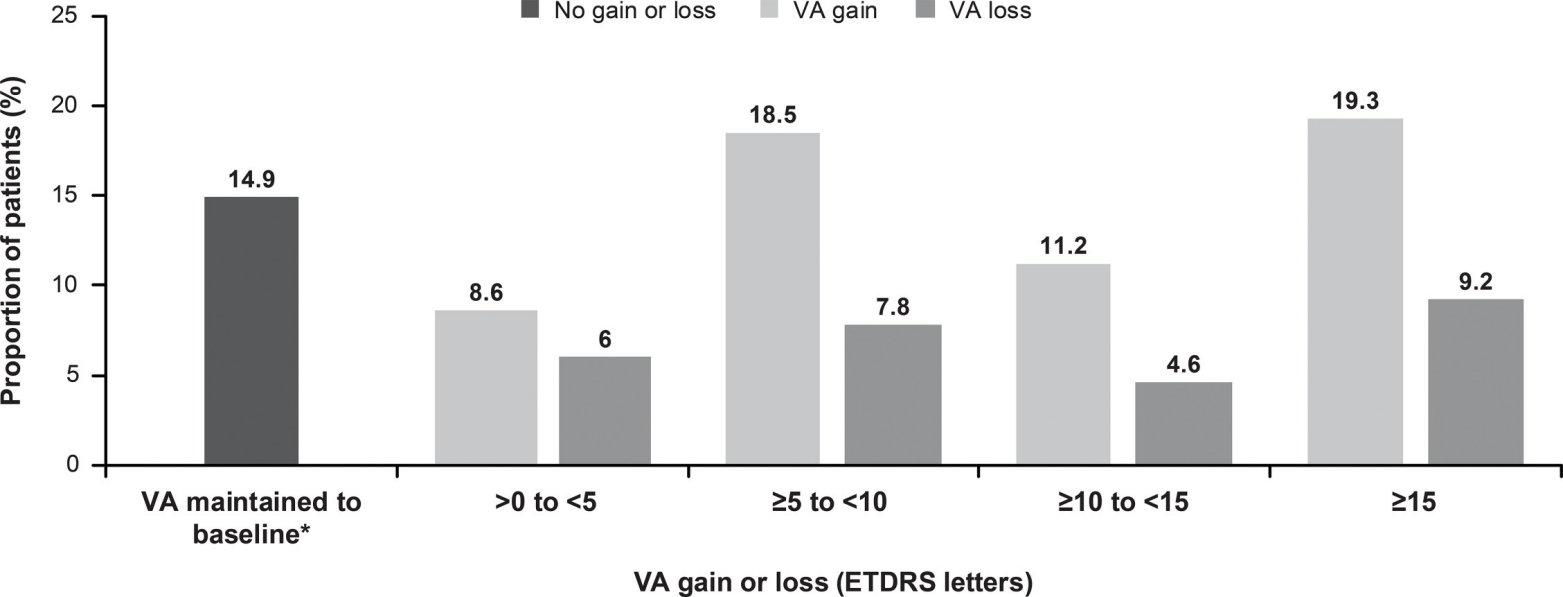
Proportion of patients with a VA gain or loss (primary treated eye set). *Includes patients with 0 letters loss. The primary treated eye set included all primary treated eyes in patients included in the safety set. The safety set consisted of patients in the enrolled set who were treated with at least one dose of ranibizumab during this study or prior to the start of the study and had at least one safety assessment after the first treatment. For treatment-naïve eyes, the date of first on-study injection with ranibizumab was considered the baseline date. For the 1-year time period, all patients with non-missing baseline VA and year 1 VA performed anywhere between Day 319 and Day 409 but who had been in the study for at least 365 days from baseline to last follow-up date were included in the analysis. ETDRS, Early Treatment Diabetic Retinopathy Study; VA, visual acuity.

At year 1, majority of the patients (57.6%; n = 289) had VA gains when treated with ranibizumab (Fig 5). At year 1, VA was maintained (0 letters loss) in 14.9% (n = 75) of patients (Fig 5). VA loss of >0 to <15 letters was reported in 18.4% of patients (n = 92), whereas 9.2% of patients (n = 46) had a VA loss of ≥15 letters (Fig 5).

### Treatment exposure and visits

**Fig 6:**
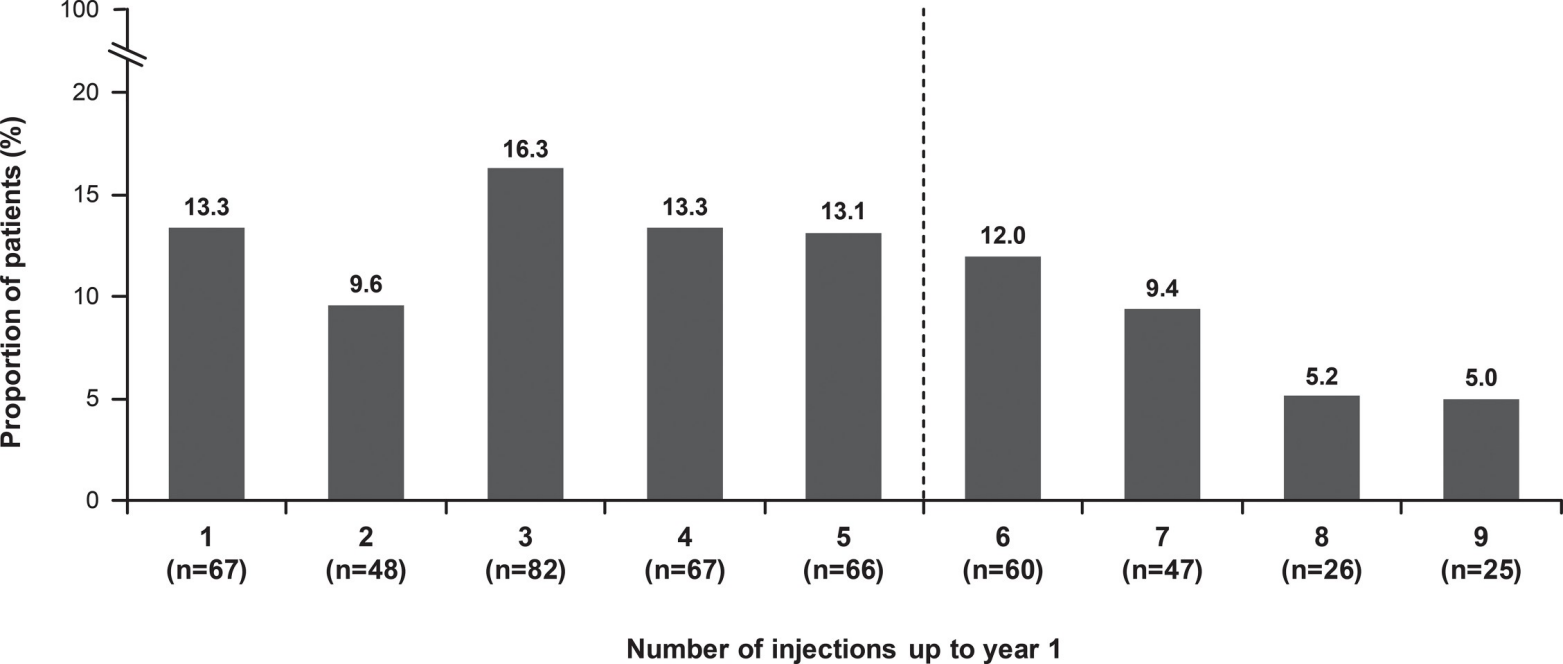
Frequency of injections over 12 months (N = 502, safety set). The safety set consisted of patients in the enrolled set who were treated with at least 1 dose of ranibizumab during this study or prior to start of study and had at least 1 safety assessment after the first treatment. 1.4% (n = 7), 1.0% (n = 5), and 0.4% (n = 2) of patients received 10, 11, and 12 injections respectively. n = number of evaluable patients with baseline and year 1 data. For treatment-naïve eyes, the date of first on-study injection with ranibizumab was considered the baseline date. For the 1-year time period, all patients with non-missing baseline VA and year 1 VA performed anywhere between Day 319 and Day 409 but who had been in the study for at least 365 days from baseline to last follow-up date were included in the analysis. VA, visual acuity.

Over 1 year, the mean (SD) number of injections and monitoring visits were 4.5 (2.5) and 8.1 (3.4), respectively. Overall, 47.5% of patients received ≥5 injections. Approximately 53% of patients received ≤4 injections in the first year of the treatment with 39.2% of patients receiving ≤3 injections (Fig 6).

### Country specific analysis of visual acuity outcomes and treatment patterns

**Fig 7:**
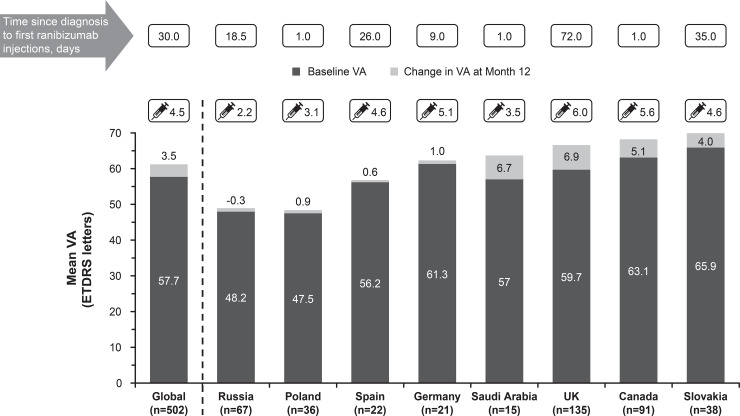
Mean change in visual acuity, baseline visual acuity, and average injection numbers across various regions (primary treated eye set). The primary treated eye set included all primary treated eyes in patients included in the safety set. Top recruiting countries (treatment-naïve DME) with highest evaluable baseline and year 1 data. For the 1-year time period, all patients with non-missing baseline VA and year 1 VA performed anywhere between Day 319 and Day 409 but who had been in the study for at least 365 days from baseline to last follow-up date were included in the analysis. DME, diabetic macular edema; ETDRS, Early Treatment Diabetic Retinopathy Study; VA, visual acuity.

The mean change in VA was similar across the countries that recruited the highest number of treatment-naïve patients with DME (Fig 7). The mean change in VA letter scores was highest in patients from the UK (+6.9) and Saudi Arabia (+6.7) with a mean of 6.0 and 3.5 ranibizumab injections, respectively. However, the mean VA gains were lower in many countries; Spain (0.6 letters), Poland (0.9 letters), and Germany (1.0 letters) with an average of 4.6, 3.1, and 5.1 injections, respectively. In Russia, there was a mean loss of 0.3 letters with a mean of only 2.2 injections. It was also observed that countries such as Canada and Slovakia with a higher baseline VA had a higher mean VA gain at year 1 whereas countries such as Russia and Poland where patients had lower baseline VA and received <4 injections (Fig 7). The median time from diagnosis of DME to first ranibizumab injection was within 26 days in all the highest recruiting countries except Slovakia and the UK where the median time was more than a month (Fig 7).

### Safety outcomes

Overall, across all treatment-naïve DME patients (N = 1,063), ocular AEs were reported in 7.2% (n = 76) of patients. The most commonly reported ocular AEs were cataract (1.6%), conjunctival hemorrhage (0.8%), macular edema (0.7%), and increased intraocular pressure and vitreous hemorrhage (each 0.6%; Table 4). The rate of non-ocular AEs was 10.1% (n = 107). The most commonly reported non-ocular AEs were influenza (0.8%), headache (0.7%), pneumonia (0.6%), myocardial infarction (0.5%), acute myocardial infarction, dyspnea, hypercholesterolemia, hypertension, and lower respiratory tract infection (each 0.4%; Table 4).

**Table 4:** Ocular (primary treated eye) and non-ocular adverse events in treatment-naïve patients with DME.

Preferred term, n (%)	Treatment-naïve DME patients (N = 1,063)
**Ocular, total**	**76 (7.2)**
Cataract	17 (1.6)
Intraocular pressure increased	6 (0.6)
Vitreous hemorrhage	6 (0.6)
Conjunctival hemorrhage	8 (0.8)
Diabetic retinopathy	3 (0.3)
Eye pain	3 (0.3)
Conjunctivitis	2 (0.2)
Macular edema	7 (0.7)
Corneal abrasion	3 (0.3)
Ocular hypertension	2 (0.2)
Vision blurred	2 (0.2)
Vitreous floaters	4 (0.4)
Retinal detachment	2 (0.2)
Diabetic retinal edema	2 (0.2)
Lacrimation increased	2 (0.2)
Eye irritation	2 (0.2)
Ocular hyperemia	2 (0.2)
Burning sensation	2 (0.2)
**Non-ocular, total**	**107 (10.1)**
Death	10 (0.9)
Lower respiratory tract infection	4 (0.4)
Myocardial infarction	5 (0.5)
Pneumonia	6 (0.6)
Hypertension	4 (0.4)
Influenza	8 (0.8)
Urinary tract infection	3 (0.3)
Anemia	2 (0.2)
Fall	3 (0.3)
Renal failure	2 (0.2)
Acute myocardial infarction	4 (0.4)
Cellulitis	2 (0.2)
Headache	7 (0.7)
Angina pectoris	3 (0.3)
Dyspnoea	4 (0.4)
Osteoarthritis	2 (0.2)
Acute kidney injury	2 (0.2)
Nasopharyngitis	2 (0.2)
Chronic kidney disease	2 (0.2)
Diabetic foot	2 (0.2)
Syncope	2 (0.2)
Dizziness	3 (0.3)
Blood pressure increased	2 (0.2)
Hypercholesterolemia	4 (0.4)
Hypothyroidism	2 (0.2)
Coronary artery disease	2 (0.2)
Confusional state	2 (0.2)
Lower limb fracture	2 (0.2)
Pulmonary embolism	2 (0.2)

Primary treated eye set for ocular AEs and safety set for non-ocular AEs.

The primary treated eye set included all primary treated eyes in patients included in the safety set.

Only SAEs occurring during the safety observation period are included.

The safety set consisted of patients in the enrolled set who were treated with at least one dose of ranibizumab during this study or prior to the start of the study and had at least one safety assessment after the first treatment.

Ocular and non-ocular AEs ≥0.2% of patients are shown.

Only AEs occurring during the safety observation period are included. A patient with multiple occurrences of a AE is counted once per preferred term. A patient with multiple AEs is counted only once in the total row. Patients with a baseline visit date present are included.

Data collected until the last recorded follow-up date were used to perform the analyses.

For treatment-naïve eyes, the date of first on-study injection with ranibizumab was considered the baseline date.

AE, adverse event; DME, diabetic macular edema.

Ocular AEs suspected to be related to ranibizumab and/or ocular injection were reported in 22 (2.1%) treatment-naïve DME patients and of these, 0.2% were suspected to be related to ranibizumab (n = 2). Overall, 5 (0.5%) patients had non-ocular AEs suspected to be related to ranibizumab and/or ocular injection and all were suspected to be related to ranibizumab.

The rate of ocular SAEs was 0.3% (n = 3). There was one case each of diabetic retinopathy, vitreous hemorrhage, ophthalmic herpes, and ulcerative keratitis. No cases of endophthalmitis were reported in treatment-naïve patients with DME (Table 5). Non-ocular SAEs were reported in 5.8% (n = 62) of treatment-naïve DME patients in the safety set. The most commonly reported non-ocular SAEs reported were myocardial infarction (0.5%), pneumonia (0.5%), and acute myocardial infarction (0.4%). There was a total of 10 deaths (0.9%) in the study; none of which were suspected to be related to the study drug. (Table 5).

**Table 5:** Ocular (primary treated eye) and non-ocular serious adverse events in treatment-naïve patients with DME.

Preferred term, n (%)	Treatment-naïve DME patients (N = 1,063)
**Ocular, total**	**3 (0.3)**
Diabetic retinopathy	1 (0.1)
Vitreous hemorrhage	1 (0.1)
Herpes ophthalmic	1 (0.1)
Ulcerative keratitis	1 (0.1)
**Non-ocular, total**	**62 (5.8)**
Death	10 (0.9)
Myocardial infarction	5 (0.5)
Pneumonia	5 (0.5)
Acute myocardial infarction	4 (0.4)
Renal failure	2 (0.2)
Acute kidney injury	2 (0.2)
Cellulitis	2 (0.2)
Chronic kidney disease	2 (0.2)
Angina pectoris	2 (0.2)
Dyspnoea	3 (0.3)
Syncope	2 (0.2)
Lower respiratory tract infection	2 (0.2)
Coronary artery disease	2 (0.2)
Pulmonary embolism	2 (0.2)
Confusional state	2 (0.2)

Primary treated eye set for ocular SAEs and safety set for non-ocular SAEs.

The primary treated eye set included all primary treated eyes in patients included in the safety set.

The safety set consisted of patients in the enrolled set who were treated with at least one dose of ranibizumab during this study or prior to the start of the study and had at least one safety assessment after the first treatment.

Only SAEs occurring during the safety observation period are included.

Non-ocular SAEs ≥0.2% of patients are shown.

There were no cases of endophthalmitis reported in the study.

A patient with multiple occurrences of a SAE is counted once per preferred term. A patient with multiple SAEs is counted only once in the total row. Patients with a baseline visit date present are included.

Data collected until the last recorded follow-up date were used to perform the analyses.

For treatment-naïve eyes, the date of first on-study injection with ranibizumab was considered the baseline date.

DME, diabetic macular edema; SAE, serious adverse event.

Ocular SAE leading to the discontinuation of ranibizumab was reported in 1 (0.1) patient. Thirty-two (3.0%) patients had non-ocular SAEs leading to discontinuation of ranibizumab.

## Discussion

LUMINOUS was a large, 5-year, multicenter, prospective, non-interventional, global study that enrolled >30,000 patients across 42 countries that assessed the effectiveness and safety of ranibizumab 0.5 mg across all approved indications in a real-life clinical setting. It is one of the first RWE studies to have enrolled more than 4,700 patients with DME with a diverse medical histories and medical comorbidities. The 1-year results show that ranibizumab treatment in treatment-naïve patients with DME led to better mean VA gains in patients who had a lower baseline VA. This was generally achieved with a relatively low mean number of injections. VA gains, however, were greater in patients receiving ≥5 ranibizumab injections during the year, particularly in those who received loading doses. The LUMINOUS study results show that good baseline VA (letter scores ≥73) can be maintained at year 1 with adequate ranibizumab treatment. It was also observed that some patients were able to achieve ≥73 letters (good vision) who had a baseline VA letter scores of <73 (poorer starting vision).

The mean VA gains observed in LUMINOUS (3.5 letters) are consistent with other RWE studies.[18–22] In the UK National Health Setting RWE study, the mean VA outcome observed was +6.6 letters with an average of 7.2 injections over 1 year[22] whereas in the UK Electronic Medical Records (EMR) Users Group study, the mean VA gain was 5.0 letters with a mean of 3.3 injections [23]. In other studies conducted across France, BOREAL-DME [18] and ETOILE [19], the mean VA gains were 7.4 letters and 5.3 letters, achieved with a mean of 5.1 and 5.8 injections, respectively. In the RWE study OCEAN-DME conducted in Germany, the mean VA gain was 4.0 letters with a mean of 5.2 injections over 1 year [21].

The mean number of injections observed in the LUMINOUS study and other RWE [18–23] DME studies was slightly lower than that observed in other RCTs [9–11]. As per the approved product label of ranibizumab in the European Union, patients with DME should receive a minimum of three ranibizumab injections until VA stabilization with further re-treatment as required, equivalent to an average of 6–7 injections in the first year, a dosing regimen that is commonly seen in robust RCTs [8]. The lower number of injections in LUMINOUS might indicate these patients with a broader heterogeneity and more varied baseline comorbidities than RCTs–were actually reasonably managed in a real-life setting.

Injection frequency plays an important role in VA gains as is evident from the real-world LUMINOUS study where the greatest VA gains were seen in patients who received ≥5 injections. These findings support the fact that at least ≥5 injections are needed to achieve optimal VA at the end of 1 year. Moreover, patients who received three loading doses at the study start had higher VA gains than those who did not and these findings are supported from the previously conducted BOREAL-DME, ETOILE, and UK-EMR studies [18, 19, 23]. Taking into consideration the different socio-economic and cultural aspects in a global RWE study like LUMINOUS, it may be possible that there was a bias in the treating the patients unlike other RWE studies which have been conducted in the same country or with same socio-economic and cultural aspect. An important factor that played a decisive role in determining the injection frequency was that a large proportion of patients included in this study (e.g. almost all patients recruited from Egypt) were paying for their own treatment, which made it difficult for many patients to continue the treatment as scheduled. The other challenge was making the patients aware of the disease, benefits of follow-up visits, and continuous treatments, as some patients did not attend follow-up visits until their vision worsened.

Real-world studies like LUMINOUS show that relative under-treatment is common for many DME patients treated with ranibizumab, as with other anti-VEGF therapies [24]. Adequate or optimal injection frequency in the first year, led to reasonable treatment outcomes. It is therefore important for clinicians to identify and address barriers to patients receiving optimal therapy—at both a clinic level and country level. Such barriers include out-of-pocket costs, difficulties in travel, work commitments, need for bilateral treatment, lack of education regarding the need for intensive therapy over a period, and multiple patient co-morbidities, which are frequent in diabetes [25]. Many of these are readily addressable; for example, moving to a same-day bilateral injection schedule reduces the treatment burden substantially for patients with bilateral DME [26].

In LUMINOUS, a considerable proportion of patients achieved ≥73 letters (good vision) from a baseline VA of <73 letters and maintained a VA of ≥73 letters (considered to be approximate minimum driving vision in the UK, and in many other countries) [22, 27] at year 1. A VA of ≥73 letters is considered to be an important factor in preserving and maintaining of VA in patients with DME as seen in RESTORE [11] and other RWE studies [18, 22]. Moreover, stability of VA is an important factor in patients with DME who are relatively younger and that is evident from other previously reported studies [23, 28, 29].

Results from the LUMINOUS study highlights the diversity in VA outcomes and treatment patterns in real-world clinical settings between countries. Countries with higher baseline VA–Saudi Arabia, the UK, Canada, and Slovakia–showed higher VA gains at 1 year with a mean of 3.5–6.0 injections. Of these countries, UK patients achieved the highest mean VA gains (6.9 letters; n = 135) possibly because of better access to treatment, owing to the fact that ranibizumab was approved in UK in 2013 and patients had easier access to public funding. In Canada, patients aged over 65 years are covered either by public or private insurance [30, 31]. In addition, since patients are not asked to pay for their treatment at baseline, most patients receive treatment on the same day of diagnosis across Canada [32]. However, there were exceptions for countries such as Spain and Germany where the baseline VA was moderately high but the patients only maintained VA with a mean of 4.6 and 5.1 injections, respectively. These findings are probably related to low patient numbers, possible country-level differences in the age of disease onset, and baseline medical comorbidities. Further, a complete medical coverage might enable more patients to be treated without additional treatment burden or any restrictions.

Countries with lower baseline VA, Russia and Poland, had the lowest VA gains or maintained VA with a mean of only 2.2 and 3.1 injections, respectively. This could be due to variability in clinical practice or referral systems. There was also a large variation in median time from diagnosis to first treatment among countries, ranging from 1 day (Saudi Arabia, Canada, and Poland) to 366.5 days (Turkey), which presumably reflects differences in healthcare systems and access to treatment in these countries. It is also possible that differences in the definition and diagnosis of DME among countries (e.g. diagnosis based on center-involving macular edema) may also contribute to this variation.

The incidence of AEs and SAEs observed in LUMINOUS was lower than that observed in the RCTs [9–11]. There were no cases of endophthalmitis reported in the study. No deaths were suspected to be related to the study drug. Overall, the safety results observed in LUMINOUS were consistent with the well-established safety profile of ranibizumab [9–11, 20, 22].

The strength of LUMINOUS is that this is the first large-scale, multi-indication, observational study of ranibizumab. The study included patients with diverse demographics and baseline characteristics, including comorbidities which may have excluded patients from RCTs. The study re-confirms a robust effectiveness and safety profile of ranibizumab 0.5 mg for the treatment of patients with DME. In LUMINOUS, despite the great comorbidities in study population, the low glycemic control of the patients (mean HbA_1c_ was 7.9), and the multi-region and multi-country nature of LUMINOUS, with varied socio-economic and cultural conditions from one country to the other, an improvement of 3.5 letters was observed. Moreover, a good safety profile, with low safety events and no endophthalmitis cases in such a large number of patients which is consistent with the well-tolerated safety profile of ranibizumab. With a lower mean number of injections in LUMINOUS compared with RCTs, results from the LUMINOUS study showed that the treatment is sustainable in any country irrespective of their socio-economic status and if the patients were treated at more regular intervals, the VA gains could have been higher.

The LUMINOUS study had certain limitations. The variable treatment schedules across regions and inclusion of difficult to treat patients resulted in variations in the outcome data. Moreover, the study had no comparator arm, so the safety and effectiveness of ranibizumab cannot be directly compared with any other interventional drugs or any other therapy. Like any other RWE studies, the appropriateness with respect to the treatment exposure is lacking in LUMINOUS, which could have been avoided if the patients were followed up regularly and treated with more injections.

In summary, ranibizumab treatment over 1 year demonstrated an overall improvement and/or maintenance in VA in treatment-naïve DME patients despite generally low injection numbers. An adequate number of injections (at least 5) and a loading dose of three initial monthly ranibizumab injections seem to be important factors in determining better VA outcomes at the end of 1 year. Timely access to care, continued treatment management, and regular follow-ups determine the prognosis of DME patients throughout different countries. There were no new safety signals and the safety results were consistent with the well-established safety profile of ranibizumab in DME as reported from previous DME studies [9–11, 20, 22]. The LUMINOUS study results therefore demonstrate the effectiveness and safety of ranibizumab for the treatment of DME and may assist retina specialists to better understand the optimal usage of ranibizumab in real-life clinical settings.

## Data sharing

Novartis is committed to sharing with qualified external researchers, access to patient-level data and supporting clinical documents from eligible studies. These requests are reviewed and approved by an independent review panel on the basis of scientific merit. All data provided is anonymized to respect the privacy of patients who have participated in the trial in line with applicable laws and regulations. This trial data availability is according to the criteria and process described on www.clinicalstudydatarequest.com. The authors to confirm they had no special access or privileges that other researchers would not have.

## Supporting information

S1 Appendix The LUMINOUS study investigators. (DOCX)

S2 Appendix List of Independent Ethics Committees or Institutional Review Boards. (PDF)

S1 File (DOCX)
