# Supplementary material for: Effectiveness and safety of ranibizumab 0.5 mg in treatment-naïve patients with diabetic macular edema: Results from the real-world global LUMINOUS study
Source: PLoS One. 2020 Jun 3;15(6):e0233595. doi: 10.1371/journal.pone.0233595 (PMC7269267; doi:10.1371/journal.pone.0233595)
Supplement: S1 Appendix — (DOCX) [file pone.0233595.s001.docx]

**S1 Appendix.** The LUMINOUS study investigators

| **Investigator** | **Address** |
| --- | --- |
| **Argentina** | |
| Dr. Alejandro Cuomo | Clinica Modelo de Lanus, Lanus, Buenos Aires, 1824, Argentina |
| Dr. Alejandro Ferrero | Clínica de Ojos Srl, Santa Fe, Santa Fe, S3000FQI, Argentina |
| Dr. Ana Brant | Clínica Privada de Ojos José León Suárez, San Martin, Buenos Aires, 1650, Argentina |
| Dr. Andres Jacofsky | Consultorio Dr. Andres Jakofsky, Buenos Aires, Buenos Aires, C1122AAI, Argentina |
| Dr. Carlos Petry | Oftalmologia Integral, Mar del Plata, Buenos Aires, B7600, Argentina |
| Dr. David Pelayes | Consultorio de Investigaciones Oftalmológicas, Buenos Aires, Buenos Aires, 1425, Argentina |
| Dr. Federico Furno Sola | Grupo laser Visión - Rosario Eximer Laser Visión, Rosario, Santa Fe, S2000DLA, Argentina |
| Dr. Gustavo Fugazzotto | Consultorios Oftalmologico Dres Fugazzotto, Guaymallén, Mendoza, M5500, Argentina Plaza Vision S.A., Mendoza, Mendoza, M5500, Argentina |
| Dr. Gustavo Russo | Hospital Oftalmológico Malvinas Argentinas, Malvinas Argentinas, Buenos Aires, 1846, Argentina |
| Dr. Hugo Sudria | Instituto Oftamológico de Córdoba SA, Cordoba, Cordoba, 5000, Argentina |
| Dr. Matko Vidosevich | Microcirugia Ocular (Clínica MICRO), Rosario, Santa Fe, S2000CTC, Argentina |
| Dr. Mauricio Martinez Cartier | Instituto de la Visión, Ciudad Autonoma Buenos Aires, Ciudad Autonoma Buenos Aires, C1122, Argentina |
| Dr. Meroni Mariano | Clínica Oftalmológica Meroni, La Plata, Buenos Aires, B1900, Argentina |
| Dr. Nelida Rosso Nano | Clínica de Ojos, Concepción del Uruguay, Entre Rios, CP 3260, Argentina |
| Dr. Noe Rivero Covre | Clínica Dr Rivero Covre, Villa Gobernador Galvez, Santa Fe, 2124, Argentina |
| Dr. Pablo Ventola | Centro Oftalmologico Ventola, Caballito, Buenos Aires, C1437AVJ, Argentina |
| Dr. Paula Donato | Instituto Donato, Ciudad Autonoma Buenos Aires, Ciudad Autonoma Buenos Aires, 1428, Argentina |
| Dr. Pedro Miranda | Dyter S.A., Mendoza, Mendoza, 5500, Argentina |
| **Australia** | |
| Dr. Alex Hunyor | Retina Associates - Chatswood Retina Service, Chatswood, New South Wales, 2067, Australia |
| Prof. Andrew Chang | Sydney Retina Eye Clinic and Day Surgery, Sydney, New South Wales, 2000, Australia |
| Dr. Anthony Dunlop | Forster Eye Surgery, Forster, New South Wales, 2428, Australia |
| Prof. Anthony Kwan | Queensland Eye Institute, South Brisbane, Queensland, 4101, Australia |
| Dr. Brendan Vote | Tasmanian Eye Institute, South Launceston, Tasmania, 7249, Australia |
| Dr. Erwin Groeneveld | Brisbane Eye Clinic, Spring Hill, Queensland, 4000, Australia |
| Prof. Grant Raymond | Eye Consultants SA, North Adelaide, 5006, Australia |
| Prof. Ian McAllister | Lions Eye Institute, Nedlands, 6009, Australia |
| Dr. Jagjit Gilhotra | Adelaide Eye and Retina Centre, Adelaide, South Australia, 5000, Australia |
| Dr. James Wong | Strathfield Retina Clinic, Sydney, New South Wales, 2135, Australia |
| Dr. Jennifer Arnold | Marsden Eye Specialists, Sydney, New South Wales, 2150, Australia |
| Dr. John Clark | St. John of God Hospital, Geelong, Victoria, 3220, Australia |
| Prof. Mark Gillies | Sydney Eye Hospital, Sydney, New South Wales, 2000, Australia |
| Dr. Mark Gorbatov | Retina and Vitreous Centre, Strathfield, New South Wales, 2135, Australia |
| Dr. Mark Steiner | Oakleigh Eye Center, Oakleigh, Victoria, 3166, Australia |
| Dr. Nitin Verma | Hobart Eye Surgeions, Hobart, 5000, Australia |
| Dr. Paul Beaumont | Retina & Vitreous Centre, Sydney, New South Wales, 2000, Australia |
| Prof. Paul Mitchell | Private Rooms, Eye Clinic (B4a) Westmead Hospital, Westmead, New South Wales, 2145, Australia |
| Dr. Robert Bourke | Vision Retinal Institute, Southport, Queensland, 4215, Australia |
| Dr. Robert Chong | Southern Ophthalmology, Sydney, New South Wales, 2217, Australia |
| Prof. Robin Guymer | Macular Research Unit, Centre for Eye Research, East Melbourne, Victoria, 3002, Australia |
| Dr. Rohan Merani | Macquarie University, Sydney, New South Wales, 2109, Australia |
| Prof. Simon Chen | Vision Eye Institute Chatswood, Chatswood, 2067, Australia |
| Dr. Vivek Phakey | Waverley Eye Clinic, Glen Waverley, Victoria, 3150, Australia |
| Dr. William Campbell | Melbourne Retina Associates, East Melbourne, Victoria, 3002, Australia |
| **Austria** | |
| Prof. Siegfried Priglinger | Prof. Dr. Siegfried Priglinger, Linz, 4020, Austria |
| Prof. Ursula Schmidt-Erfurth | General University Hospital of Vienna, Vienna, Vienna, 1090, Austria |
| **Belgium** | |
| Prof. Anita Leys | Academic Hospital St. Rafaël, Leuven, 3000, Belgium |
| Dr. Danielle Vangermeersch | Imeldaziekenhuis, Bonheiden, 2820, Belgium |
| Prof Elisabeth Van Aken | Sint-Elisabeth Ziekenhuis, Zottegem, 9620, Belgium |
| Dr. Els Mangelschots | Virga Jesseziekenhuis, Hasselt, 3500, Belgium |
| Dr. Filip Mergaerts | RZ Tienen Campus St Elisabeth Aarschot, Aarschot, 3201, Belgium |
| Dr. Florence Rasquin | Hôpital Erasme, Bruxelles, 1070, Belgium |
| Dr. Guy Sallet | Ooginstituut, Aalst, 9300, Belgium |
| Dr. Ingrid Van der Donck | General Hospital Heilige Familie, Aarschot, 2840, Belgium |
| Dr. Isabelle Verhaeghe | General Hospital Maria Middelares, Ghent, 9000, Belgium |
| Dr. Jan Van Looveren | UZ Antwerpen, Edegem, 2650, Belgium |
| Dr. Laurent Levecq | Cliniques Universitaires de Mont-Godinne, Yvoir, B-5530, Belgium |
| Dr. Luana Bistreanu | Centre Hospitalier de Mouscron, Mouscron, 7700, Belgium |
| Dr. Stefano Barile | Centre Hospitalier Régional de Namur, Namur, 5000, Belgium |
| Dr. Thierry Vandercam | Centre Hospitalier de Dinant, Dinant, 5500, Belgium |
| **Brazil** | |
| Dr. Aderbal de Albuquerque Alves Junior | HFSE - Hospital Federal dos Servidores do Estado do Rio de Janeiro, Rio de Janeiro, 20221, Brazil |
| Dr. Arnaldo Bordon | ANGIOCORPORE, Sao Paulo, 040 21, Brazil |
| Dr. Danielle Lavinsky | Clínica Lavinsky Oftalmologia, Porto Alegre, Rio Grande do Sul, 90440, Brazil |
| Dr. Marcio Nehemy | Instituto da Visão, Belo Horizonte, Minas Gerais, 30150-270, Brazil |
| Dr. Marcos Pereira de Avila | CBCO - Centro Brasileiro de Cirurgia de Olhos, Goiânia, Goiás, 74210, Brazil |
| Dr. Osias de Souza | Centro Medico de Oftalmologia, Cambui, Campinas, 13092, Brazil |
| **Canada** | |
| Dr. Arif Samad | Ophthalmology Private Practice Dr Samad, Halifax, Nova Scotia, B3H 1Y6, Canada |
| Dr. Berhard Hurley | The Ottawa Hospital - General Campus, University of Ottawa Eye Institute, Ottawa, Ontario, K1H 8L6, Canada |
| Dr. David Chow | Toronto Retina Institute, Don Mills, Ontario, M1E 4A7, Canada |
| Dr. Deepa Yoganathan | North Toronto Eye Care, Downsview, Ontario, M3N 2V6, Canada |
| Dr. Don Nixon | Trimed Eye Center, Barrie, Ontario, L4M 4S5, Canada |
| Dr. Geoff Williams | Calgary Retina Consultants, Calgary, Alberta, T2H 0C8, Canada |
| Dr. Jason Noble | Scarborough Eye Clinic, Scarborough, Ontario, M1R 3A6, Canada |
| Dr. Laurent Lalonde | Institut de loeil des Laurentides, Boisbriand, Quebec, J7H 1S6, Canada |
| Dr. Luis Riveros | Miramichi Regional Hospital, Miramichi, New Brunswick, E1V 1N9, Canada |
| Dr. Michael Brent | Toronto Western Hospital, Toronto, Ontario, M5T 2S8, Canada |
| Dr. Murray Erasmus | Retina Consultants of Victoria (RCV), Victoria, British Columbia, V8V 4X3, Canada |
| Dr. Patrick Saurel | Clinique Dr. Patrick Saurel, Drummondville, Quebec, J2C 2C4, Canada |
| Dr. Raman Tuli | The Retina Centre of Ottawa, Ottawa, Ontario, K1Z 8R2, Canada |
| Dr. Sanjay Sharma | The Macula Clinic at the Wedgewood, Brockville, Ontario, K6V 0A6, Canada The Macula Clinic at the Bayview Medical Clinic, Belleville, Ontario, K8N 1E6, Canada |
| Dr. Sohel Somani | EyeMD Institute Suite, Brampton, Ontario, L6Y 0P6, Canada |
| Dr. Steve Dorrepaal | Clarity Eye Institute, Vaughan, Ontario, L4K 0C5, Canada |
| Dr. Thomas Sheidow | SJHC St. Joseph's Hospital, London, Ontario, N6A 4V2, Canada |
| Dr. Vineet Arora | Vineet I Arora Medicine Professional Corporation, Hamilton, Ontario, L9C 5R3, Canada |
| **Chile** | |
| Dr. Alejandro Lutz Herrerra | Hospital del Cobre Salvador Allende Gossens, Calama, 1399001, Chile |
| **China** | |
| Prof. Fang Wang | No. 10 People's Hospital of Shanghai, Shanghai, 200072, China |
| Prof. Fangtian Dong | Peking Union Medical College Hospital, Beijing, 100032, China |
| Prof. Gezhi Xu | Eye and ENT hospital, Shanghai, 200000, China |
| Prof. Guanfang Su | No.2 Hospital Affiliated to Jilin University, Changchun City, Jilin, 130041, China |
| Prof. Guoji Wu | Xiamen Eye Centre, Fujian, 361001, China |
| Dr. Hong Dai | Beijing Hospital, Beijing, 100730, China |
| Prof. Hongsheng Bi | Shierming Eye Hospital, Jinan, Shandong, 250001, China |
| Prof. Libo Xiao | Yunnan 2nd People's Hospital, Kunming, Yun'nan, 650021, China |
| Prof. Lin Lu | Zhongshan Ophthalmic Center, Sun Yat-sen University, Guangzhou, Guangdong, 510060, China |
| Prof. Liu Yang | Peking University First Hospital, Beijing,P.R., Beijing, 100034, China |
| Prof. Peiquan Zhao | Xinhua Hospital Affiliated to Shanghai Jiao Tong University School of Medicine, Shanghai, 200092, China |
| Prof. Wenbin Wei | Beijing Tong Ren Hospital, Capital Medical University, Beijing, 100730, China |
| Prof. Xian Wang | The Affiliated Hospital of Guiyang Medical College, Guiyang, Guizhou, 500000, China |
| Prof. Xiaorong Li | Tianjin Medical University Eye Center, Tianjin, 300384, China |
| Prof. Xiaowei Gao | No. 474 Hospital of PLA, Wulumuqi, Xinjiang Uygur, 830000, China |
| Prof. Xun Xu | Shanghai First People's Hospital, Shangai, 200080, China |
| Prof. Yi Wang | Southwest Hospital, Chongqing, Chongqing, 400038, China |
| Prof. Yiqiao Xing | Renmin Hospital of Wuhan University, Wuhan, Hubei, 430060, China |
| Prof. Zhengqin Yin | Chinese PLA General Hospital, Beijing, Beijing, 100853, China |
| Prof. Zhizhong Ma | Peking University Third Hospital, Beijing, 100191, China |
| **Colombia** | |
| Dr. Andres Amaya | Sociedad de Cirugía Ocular, Bogotá, , Colombia |
| Dr. Carlos Velez | Clínica Oftalmológica del Atlántico, Barranquilla, , Colombia |
| Dr. Diego Fernando Paipilla | Optisalud SAS, Yopal, , Colombia |
| Dr. Javier Andres Bernal Urrego | Clinica Oftalmologica, Armenia, , Colombia |
| Dr. Juan Pablo Sinisterra | Instituto de Ciegos y Sordos INSORP, Cali, Colombia |
| Dr. Maria Teresa Bernal | Clínica Barraquer, Bogotá, , Colombia |
| **Costa Rica** | |
| Dr. Lihteh Wu | Instituto de Cirugia Ocular, San José, San José, Costa Rica |
| Dr. Manrique Ortiz | Oftalmocima, San José, San José, Costa Rica |
| Dr. Teodoro Evans | Clínica 20/20, San José, San José, Costa Rica |
| **Czech Republic** | |
| Dr. Hana Fidranska | Fakultni nemocnice Plzen, Plzen, 323 00, Czech Republic |
| Dr. Jan Ernest | Ustredni vojenska nemocnice Praha, Praha, 169 02, Czech Republic |
| Dr. Jan Nemcansky | University Hopsital Ostrava, Ostrava, 708 52, Czech Republic |
| Dr. Jan Studnicka | Fakultni nemocnice Hradec Kralove, Hradec Kralove, 500 05, Czech Republic |
| Dr. Jiri Rehak | Fakultni nemocnice Olomouc, Olomouc, 775 20, Czech Republic |
| Dr. Martina Zavorkova | Krajska zdravotni, a.s. - Masarykova nemocnice v Usti nad Labem, Usti nad Labem, 40113, Czech Republic |
| Dr. Miroslav Veith | Fakultni nemocnice Kralovske Vinohrady, Praha 10, 100 34, Czech Republic |
| Prof. Petr Kolar | Fakultni nemocnice Brno, Brno, 625 00, Czech Republic |
| Dr. Zora Dubska | Charles University Hopsital 1st Faculty of Medicine, Praha 2, 128 08, Czech Republic |
| **Dominican Republic** | |
| Dr. Niurka Leonor | Centro Cardio-Neuro Oftalmológico y Trasplante (CECANOT), Santo Domingo, Santo Domingo, Dominican Republic |
| Dr. Rosina Negrin | Consultorio Oftalmológico Medicalnet, Santo Domingo, Santo Domingo, Dominican Republic |
| **Ecuador** | |
| Dr. Mario Polit Macias | Centro Medico Quirurgico Oftalmologico Alta Vision, Guayaquil, 090 150, Ecuador |
| **Egypt** | |
| Dr. Islam Waly | Egypt Air Hospital, Cairo, Cairo, Egypt |
| Prof. Mohamed Mahgoub | Eye Subspeciality Center, Cairo, Cairo, Egypt |
| Prof. Noha Khater | Al Mouneer Diabetic Retina Center, Giza, Cairo, Egypt |
| Dr. Walid Sheta | Alex Eye Center, Alexandria, Alexandria, Egypt |
| Prof. Yasser Soliman | Al Rowad Hospital, Giza, Cairo, Egypt |
| **France** | |
| Dr. Alain Donati | Cabinet d'Ophtalmologie, Melun, Seine et Marne, 77000, France |
| Prof. Catherine Creuzot-Garcher | CHU Dijon - Hopital General, Dijon, Côte-d'Or, 21000, France |
| Dr. Catherine Favard | Centre Ophtalmologique de L'Odeon, Paris, 75006, France |
| Dr. Christian Delhay | Clinique Ocean, Vannes, Côte-d'Or, 56000, France |
| Prof. Eric Souied | Centre Hospitalier Intercommunal de Créteil, Creteil, Val de Marne, 94010, France |
| Dr. Franck Rumen | Visiopole Private Practice, Lagord, Charente, 17140, France |
| Prof Gabriel Coscas | Cabinet Odeon , Paris, 75006, France |
| Dr. Hassiba Oubraham | Clinique de Montargis, Montargis, Loiret, 45200, France |
| Prof. Jean-Francois Korobelnik | Groupe Hospitalier Pellegrin -Hôpital Pellegrin, Bordeaux, Gironde, 33000, France |
| Dr. Laurence Mahieu | CHU Toulouse, Hôpital Paule de Vignier, Toulouse, Cedex 9, 31059, France |
| Dr. Laurent Khaitrine | Polyclinique de Courlancy, Reims, Marne, 51100, France |
| Prof. Laurent Kodjikian | Centre Hospitalier de la Croix Rousse, Lyon, Rhone, 69317, France |
| Dr. Martine Faysse | Fondation Ophtalmologique Adolphe de Rothschild, Paris, 75019, France |
| Prof. Michel Weber | CHU Nantes - Hôtel Dieu, Nantes Cedex 1, Loire Atlantique, 44093, France |
| Dr. Ramin Tadayoni | Hôpital Lariboisière, Paris, 75475, France |
| Dr. Salomon-Yves Cohen | Centre Ophtalmologique d’Imagerie et de Laser, Paris, Val de Marne, 75015, France |
| Dr. Stephanie Baillif-Gostoli | CHU de Nice - Hôpital Lenval, Nice, Alpes Maritimes, 060 06, France |
| Dr. Vincent Gualino | Clinique Orl Honore Cave, Montauban, Tarn et Garonne, 82000, France |
| **Germany** | |
| Dr. Annette Grote-Schmidt | Private practice_ Dr Grote-Schmidt, Tönisvorst, Nordrhein Westfalen, 47918, Germany |
| Dr. Arthur Mueller | Klinikum Augsburg Augenklinik, Augsburg, Bayern, 86156, Germany |
| Dr. Barbara Fuchs-Koelwel | Dr. med. Claus Fuchs Fachärzte für Augenheilkunde, Regensburg, Bayern, 93047, Germany |
| Dr. David Schell | Augenlaserzentrum, Neu-Ulm, Bayern, 89231, Germany |
| Dr. Erik Beeke | Klinikum Osnabrück, Osnabrück, Niedersachsen, 49076, Germany |
| Dr. Eva-Maria Weinschrod | Fachärztin für Augenheilkunde, Rothenburg, Bayern, 91541, Germany |
| Prof. Focke Ziemssen | Eberhard Karls University Eye Hospital, Tuebingen, Baden Wuerttemberg, 72076, Germany |
| Prof. Frank Holz | University of Bonn, Bonn, Nordrhein Westfalen, 53127, Germany |
| Dr. Georg Spital | St. Franziskus Hospital, Muenster, Nordrhein Westfalen, 48145, Germany |
| Dr. Gunther Kahle | Praxis Arzt für Augenheilkunde, Berlin, 10707, Germany |
| Dr. Kathleen Steinberg | Praxis Steinberg, Berlin, 10367, Germany |
| Dr. Mark Steiner | Augenklinik Dannenberg, Dannenberg Elbe, Niedersachsen, 29451, Germany |
| Prof. Nicole Eter | Universitaetsklinikum Muenster, Münster, Nordrhein Westfalen, 48149, Germany |
| Dr. Peter Kaupke | Praxis_Dr Kaupke, Hamburg, 22587, Germany |
| Prof. Sascha Fauser | Universitaetsklinikum Koeln, Köln, Nordrhein Westfalen, 50924, Germany |
| Dr. Steffen Rabethge | Dr. Rabethge Klinik GmbH, Schriesheim, Baden Wuerttemberg, 69198, Germany |
| Dr. Susanne Kaskel-Paul | Klinikum Lüdenscheid, Lüdenscheid, Nordrhein Westfalen, 58515, Germany |
| Dr. Thomas Grasbon | Augenarztpraxis Grasbon, Ingolstadt, Bayern, 85049, Germany |
| **Greece** | |
| Dr. Aleksandros Charonis | Athens Vision Eye Institute, Athens, 17673, Greece |
| Dr. Aliki Liaska | General Hospital of Lamia, Lamia, 35100, Greece |
| Dr. Efstratios Parikakis | Ophtalmiatrio Athinon - Athens Eye Hospital, Athens, 10672, Greece |
| Dr. Emmanouil Christodoulakis | General Hospital of Rethymnon, Rethymnon, 74100, Greece |
| Prof. Evangelia Tsironi | University General Hospital of Larissa, Larissa, 41110, Greece |
| Dr. Ioannis Datseris | Omma Ophtalmological Institute of Athens, Athens, 11525, Greece |
| Dr. Miltiadis Tsilimbaris | University Eye Hospital of Heraklion, Heraklion, 71201, Greece |
| Prof. Nicolas Farmakakis | University Hopsital of Patras, Patras, 26504, Greece |
| Dr. Stamatia Xirou | Red Cross Hospital -2nd Clinic, Athens, 11526, Greece |
| Prof. Stavros Dimitrakos | General Hospital Papageorgiou, Thessaloniki, 56429, Greece |
| Prof. Vasilios Kozobolis | University General Hospital of Alexandroupolis, Alexandroupolis, 68100, Greece |
| Dr. Vaso Konstantinidou | Eye Hospital of Athens -1st Clinic, Athens, 10672, Greece |
| **Guatemala** | |
| Dr. Fernando Noriega | Clínica Oftalmológica Santa Clara, Guatemala, Guatemala, Guatemala |
| **Hong Kong** | |
| Dr. Timothy Lai | Hong Kong Eye Hospital, Kowloon, Hong Kong |
| **Hungary** | |
| Dr. Andras Berta | Debreceni Egyetem Klinikai Kozpont, Debrecen, 4032, Hungary |
| Dr. Andras Seres | Budapest Retina Associates, Budapest, 1133, Hungary |
| Dr. Andrea Facsko | Szegedi Tudomanyegyetem Szent-Gyorgyi Albert Klinikai Kozpont, Szeged, 6720, Hungary |
| Dr. Janos Nemeth | Semmelweis Egyetem, Budapest, 1085, Hungary |
| Dr. Zsolt Biro | Pecsi Tudomanyegyetem, Pecs, 7624, Hungary |
| **India** | |
| Dr. Aditya Kelkar | National Institute of Ophthalmology, Pune, Maharashtra, 411005, India |
| Dr. Alay Banker | Bankers Eye Institute, Ahmedabad, Gujarat, 380009, India |
| Dr. Narendran Venkatapathy | Aravind  Eye Hospital, Coimbatore, Tamilnadu, 641014, India |
| Dr. Raja Narayanan | L. V. Prasad Eye Institute, Hyderabad, Andhra Pradesh, 500034, India |
| Dr. Taraprasad Das | L. V. Prasad Eye Institute, Bhubaneswar, Orissa, 751024, India |
| Dr. Tarun Sharma | Sankara Nethralaya, Chennai, Tamilnadu, 600006, India |
| Dr. Yograj Sharma | All India Institute of Medical Sciences, New Delhi, Delhi, 110029, India |
| **Ireland** | |
| Mr. David Keegan | Mater Private Hospital, Dublin, Dublin, 7, Ireland |
| Dr. Emer Henry | Waterford Regional Hospital, Waterford, X91, Ireland |
| Dr. Mark Cahill | Beacon Clinic, Dublin, 18, Ireland |
| Prof. Stephen Beatty | Whitfield Clinic, Waterford, X91, Ireland |
| **Israel** | |
| Prof. Hanna Garzozi | Bnai Zion Medical Center, Haifa, 31048, Israel |
| Dr. Irit Rosenblatt | Rabin Medical Center-Beilinson Campus, Petach Tikva, 4941492, Israel |
| Dr. Michaella Goldstein | Tel Aviv Sourasky Medical Center, Tel Aviv, 64239, Israel |
| **Italy** | |
| Prof. Angelo Minnella | Policlinico Universitario Agostino Gemelli, Roma, 001 68, Italy |
| Dr. Bruno Falcomata | Azienda Ospedaliera Bianchi Melacrino Morelli, Reggio Calabria, 89100, Italy |
| Dr. Daniela Dolcino | Reparto diOculistica dell Ospedale SS Antonio e Biagio e Cesare Arrigo de Alessandria, Alessandria, 15121, Italy |
| Prof. Francesco Semeraro | Azienda Socio Sanitaria Territoriale degli Spedali Civili di Brescia (Presidio Spedali Civili), Brescia, 25123, Italy |
| Dr. Luigina Tollot | Ospedale San Martino di Belluno, Belluno, 32100, Italy |
| Prof. Marco Nardi | Azienda Ospedaliero Universitaria Cisanello, Pisa, 56126, Italy |
| Prof. Maurizio Fossarello | Ospedale S.Giovanni di Dio, Cagliari, 091 00, Italy |
| Dr. Rosalia Giustolisi | Umberto I Pol. di Roma-Universita di Roma La Sapienza, Roma, 001 61, Italy |
| Dr. Tommaso Micelli Ferrari | Ente Ecclesiastico Ospedale Generale Regionale F Miulli, Acquaviva delle Fonti, Bari, 70021, Italy |
| **Japan** | |
| Dr. Akira Arakawa | Yokohama City University Medical Center, Yokohama-shi, Kanagawa-Ken, 232-0024, Japan |
| Dr. Akira Obana | Seirei Hamamatsu General Hospital, Hamamatsu-shi, Shizuoka-Ken, 430-8558, Japan |
| Dr. Akiteru Kawahara | University of Miyazaki Hospital, Miyazaki-shi, Miyazaki-Ken, 889-1692, Japan |
| Prof. Atsushi Hayashi | Toyama University Hospital, Toyama-shi, Toyama-Ken, 930-0194, Japan |
| Dr. Atsushi Hirota | Hirota Eye Clinic, Shunan-shi, Yamaguchi-Ken, 745-0017, Japan |
| Dr. Atsushi Otani | Japan Red Cross Society Wakayama Medical Center, Wakayama-shi, Wakayama-Ken, 640-8558, Japan |
| Prof. Ayame Annabel Okada | Kyorin University Hospital, Mitaka-shi, Tokyo-To, 181-8611, Japan |
| Dr. Chieko Shiragami | Kagawa University Hospital, Kita-gun, Kagawa-Ken, 761-0793, Japan |
| Dr. Chota Matsumoto | Kindai University Hospital, Osakasayama-shi, Osaka-Fu, 589-8511, Japan |
| Dr. Daisuke Jin | Akita University Hospital, Akita-shi, Akita-Ken, 010-8543, Japan |
| Dr. Eiichi Sato | Asahikawa Medical University Hospital, Asahikawa-shi, Hokkaido, 078-8510, Japan |
| Dr. Eriko Matsushita | Kochi Medical School Hospital, Nankoku-shi, Kochi-Ken, 783-8505, Japan |
| Dr. Futoshi Ishikawa | Sapporo Medical University Hospital, Sapporo-shi, Hokkaido, 060-8543, Japan |
| Prof. Goji Tomita | Toho University Ohashi Medical Center, Meguro-ku, Tokyo-To, 153-8515, Japan |
| Dr. Goro Watanabe | Koyokai Yayoi Hospital, Toyohashi-shi, Aichi-Ken, 441-8106, Japan |
| Dr. Hajime Sato | JOHAS Tohoku Rosai Hospital, Sendai-shi, Miyagi-Ken, 981-8563, Japan |
| Dr. Harumi Wakiyama | Japanese Red Cross Nagasaki Genbaku Hospital, Nagasaki-shi, Nagasaki-Ken, 852-8511, Japan |
| Dr. Hidenori Takahashi | Jichi Medical University Hospital, Shimotsuke-shi, Tochigi-Ken, 329-0498, Japan |
| Prof. Hidetoshi Yamashita | Yamagata University Hospital, Yamagata-shi, Yamagata-Ken, 990-9585, Japan |
| Dr. Hideyasu O | Hyogo Prefectural Amagasaki General Medical Center, Amagasaki-shi, Hyogo-Ken, 660-8550, Japan |
| Dr. Hiroaki Kobayashi | Juntendo University Hospital, Bunkyo-ku, Tokyo-To, 113-8431, Japan |
| Dr. Hiroaki Ushida | Shizuoka Saiseikai General Hospital, Shizuoka-shi, Shizuoka-Ken, 422-8527, Japan |
| Dr. Hiroko Imaizumi | Sapporo City General Hospital, Sapporo-shi, Hokkaido, 060-8604, Japan |
| Dr. Hisashi Matsubara | Mie University Hospital, Tsu-shi, Mie-Ken, 514-8507, Japan |
| Dr. Ichiro Ota | Shozankai Miyake Eye Hospital, Nagoya-shi, Aichi-Ken, 462-0825, Japan |
| Dr. Jiro Kogo | St. Marianna University School of Medicine Hospital, Kawasaki-shi, Kanagawa-Ken, 216-8511, Japan |
| Dr. Jun Yamada | Meiji University of Integrative Medicine, Nantan-shi, Kyoto-Fu, 629-0392, Japan |
| Prof. Kanji Takahashi | Kansai Medical University Hospital, Hirakata-shi, Osaka-Fu, 573-1191, Japan |
| Dr. Kaori Sayanagi | Osaka University Hospital, Suita-shi, Osaka-Fu, 565-0871, Japan |
| Dr. Kazuaki Nishijima | Nishijima Eye Clinic, Kyoto-shi, Kyoto-Fu, 604-0837, Japan |
| Dr. Kazuhiko Dannoue | Dannoue Eye Clinic, Kawasaki-shi, Kanagawa-Ken, 211-0053, Japan |
| Dr. Kazunori Miyata | Meiwakai Miyata Ophthalmic Hospital, Miyakonojo-shi, Miyazaki-Ken, 885-0051, Japan |
| Dr. Kohei Ishikawa | Ishikawa Eye Clinic, Shizuoka-shi, Shizuoka-Ken, 420-0841, Japan |
| Prof. Koichi Ohta | Matsumoto Dental University Hospital, Shiojiri-shi, Nagano-Ken, 399-0781, Japan |
| Dr. Koji Aoyagi | Aoyagi Eye Clinic, Ueda-shi, Nagano-Ken, 386-0002, Japan |
| Dr. Kunihiro Musashi | Musashi Dream Eye Clinic, Osaka-shi, Osaka-Fu, 543-0027, Japan |
| Dr. Masahiko Shimura | Tokyo Medical University Hachioji Medical Center, Hachioji-shi, Tokyo-To, 193-0998, Japan |
| Dr. Masahiro Miura | Tokyo Medical University Ibaraki Medical Center, Inashiki-gun, Ibaraki-Ken, 300-0395, Japan |
| Prof. Masahito Ohji | Shiga University of Medical Science Hospital, Otsu-shi, Shiga-Ken, 520-2192, Japan |
| Dr. Masaru Inatani | University of Fukui Hospital, Yoshida-gun, Fukui-Ken, 910-1193, Japan |
| Prof. Masayuki Horiguchi | Fujita Health University Hospital, Toyoake-shi, Aichi-Ken, 470-1192, Japan |
| Dr. Michiko Takamiya | Yonezawa City Hospital, Yonezawa-shi, Yamagata-Ken, 992-8502, Japan |
| Dr. Misa Suzuki | Totsukaekimae Suzuki Eye Clinic, Yokohama-shi, Kanagawa-Ken, 244-0003, Japan |
| Dr. Mizuki Tagami | Kobe Kaisei Hospital, Kobe-shi, Hyogo-Ken, 657-0068, Japan |
| Dr. Nagahisa Yoshimura | Kyoto University Hospital, Kyoto-shi, Kyoto-Fu, 606-8507, Japan |
| Prof. Nahoko Ogata | Nara Medical University Hospital, Kashihara-shi, Nara-Ken, 634-8522, Japan |
| Dr. Naotaka Kanda | Jusendo General Hospital, Koriyama-shi, Fukushima-Ken, 963-8585, Japan |
| Dr. Naoyasu Umeda | Fukuoka University Hospital, Fukuoka-shi, Fukuoka-Ken, 814-0180, Japan |
| Dr. Rei Ito | Juntendo University Urayasu Hospital, Urayasu-shi, Chiba-Ken, 279-0021, Japan |
| Dr. Rumiko Hara | Kakogawa City West Hospital, Kakogawa-shi, Hyogo-Ken, 675-8611, Japan |
| Dr. Ryo Eguchi | Ando Eye Clinic, Ashigarakami-gun, Kanagawa-Ken, 258-0003, Japan |
| Dr. Ryoichi Sugawara | Kitami Red Cross Hospital, Kitami-shi, Hokkaido, 090-8666, Japan |
| Dr. Ryoko Okayama | Saiando Ochanomizu Inoue Eye Clinic, Chiyoda-ku, Tokyo-To, 101-0062, Japan |
| Dr. Ryoko Osawa | Kaiya Ophthalmology, Hamamatsu-shi, Shizuoka-Ken, 430-0903, Japan |
| Dr. Ryusaburo Mori | Nihon University Hospital, Chiyoda-ku, Tokyo-To, 101-8309, Japan |
| Dr. Sakura Sato | Taidokai Sato Ganka Iin Domachi Clinic, Yamagata-shi, Yamagata-Ken, 990-0051, Japan |
| Dr. Santoshi Takeuchi | Yokosuka Kyosai Hospital, Yokohama-shi, Kanagawa-Ken, 236-0004, Japan |
| Dr. Shigeki Tagawa | Tagawa Eye Clinic, Kanazawa-shi, Ishikawa-Ken, 920-1151, Japan |
| Dr. Shigeki Yamanishi | Matsuyama Red Cross Hospital, Matsuyama-shi, Ehime-Ken, 790-8524, Japan |
| Dr. Shigeru Hoshiai | Hoeikai Hoshiai Eye Clinic, Saitama-shi, Saitama-Ken, 336-0963, Japan |
| Dr. Shigeto Fujimura | Kanazawa University Hospital, Kanazawa-shi, Ishikawa-Ken, 920-8641, Japan |
| Dr. Shin Yoneya | Saitama Medical University Hospital, Iruma-gun, Saitama-Ken, 350-0495, Japan |
| Dr. Shinichiro Otani | Meiwakai Kagoshima Miyata Ophthalmic Hospital, Kagoshima-shi, Kagoshima-Ken, 890-0046, Japan |
| Dr. Shinichiro Yoshida | Yoshida Eye Hospital, Hakodate-shi, Hokkaido, 041-0851, Japan |
| Dr. Shoji Kishi | Gunma University Hospital, Maebashi-shi, Gunma-Ken, 371-8511, Japan |
| Dr. Tadayuki Nishide | Yokohama City University Hospital, Yokohama-shi, Kanagawa-Ken, 236-0004, Japan |
| Dr. Taiichi Hikichi | Shuhokai Ohtsuka Eye Hospital, Sapporo-shi, Hokkaido, 001-0016, Japan |
| Prof. Taiji Sakamoto | Kagoshima University Medical And Dental Hospital, Kagoshima-shi, Kagoshima-Ken, 890-8520, Japan |
| Dr. Takahiro Uda | Ehime University Hospital, Toon-shi, Ehime-Ken, 791-0295, Japan |
| Dr. Takashi Fujishiro | Saitama Red Cross Hospital, Saitama-shi, Saitama-Ken, 338-8553, Japan |
| Prof. Takashi Kitaoka | Nagasaki University Hospital, Nagasaki-shi, Nagasaki-Ken, 852-8501, Japan |
| Dr. Takatomo Miyake | Ogaki Tokushukai Hospital, Ogaki-shi, Gifu-Ken, 503-0015, Japan |
| Prof. Taktoshi Maeno | Toho University Sakura Medical Center, Sakura-shi, Chiba-Ken, 285-8741, Japan |
| Dr. Taku Ogura | Japanese Red Cross Society Suwa Hospital, Suwa-shi, Nagano-Ken, 392-8510, Japan |
| Dr. Tatsuo Nagata | University of Occupational and Environmental Health Hospital, Kitakyushu-shi, Fukuoka-Ken, 807-8556, Japan |
| Prof. Tatsuro Ishibashi | Kyushu University Hospital, Higashi-ku, Fukuoka, 812-8582, Japan |
| Dr. Tatsushi Kaga | JCHO Chukyo Hospital, Nagoya-shi, Aichi-Ken, 457-8510, Japan |
| Prof. Tetsuju Sekiryu | Fukushima Medical University Hospital, Fukushima-shi, Fukushima-Ken, 960-1295, Japan |
| Prof. Tetsuro Oshika | Tsukuba University Hospital, Tsukuba-shi, Ibaraki-Ken, 305-8576, Japan |
| Dr. Tomohiro Iida | Tokyo Women's Medical University Hospital, Shinjuku-ku, Tokyo-To, 162-8666, Japan |
| Prof. Tomohiro Ikeda | Hyogo College of Medicine Hospital, Nishinomiya-shi, Hyogo-Ken, 663-8501, Japan |
| Dr. Tomoki Sakuraba | Aomori Prefectural Central Hospital, Aomori, Aomori, 030-8553, Japan |
| Dr. Tomoko Kawamura | Steel Memorial Hirohata Hospital, Himeji-shi, Hyogo-Ken, 671-1122, Japan |
| Dr Toru Nakazawa | Tohoku University Hospital, Sendai-shi, Miyagi-Ken, 980-8574, Japan |
| Prof. Toshihiko Ohta | Juntendo University Shizuoka Hospital, Izunokuni-shi, Shizuoka-Ken, 410-2295, Japan |
| Dr. Toshiyuki Yokoyama | Juntendo University Nerima Hospital, Nerima-ku, Tokyo-To, 177-8521, Japan |
| Dr. Toyohisa Yoshizawa | Jigankai Sanjo Eye Clinic, Sanjo-shi, Niigata-Ken, 955-0852, Japan |
| Dr. Tsuyoshi Otsuji | Kansai Medical University Takii Hospital, Moriguchi-shi, Osaka-Fu, 570-8507, Japan |
| Dr. Yasuki Ito | Nagoya University Hospital, Nagoya-shi, Aichi-Ken, 466-8560, Japan |
| Dr. Yoichi Sakurada | University of Yamanashi Hospital, Chuo-shi, Yamanashi-Ken, 409-3898, Japan |
| Dr. Yoshiaki Kiuchi | Hiroshima University Hospital, Hiroshima-shi, Hiroshima-Ken, 734-8551, Japan |
| Dr. Yoshihiro Hashimoto | Shinseikai Toyama Hospital, Imizu-shi, Toyama-Ken, 939-0243, Japan |
| Dr. Yoshihiro Wakabayashi | Tokyo Medical University Hospital, Shinjuku-ku, Tokyo-To, 160-0023, Japan |
| Prof. Yoshinori Mitamura | Tokushima University Hospital, Tokushima-shi, Tokushima-Ken, 770-8503, Japan |
| Dr. Yoshiyuki Kondo | Infinity Medical Group Kondo Eye Clinic, Hachioji-shi, Tokyo-To, 192-0081, Japan |
| Dr. Yukihiko Shiraki | Aichi Medical University Hospital, Nagakute-shi, Aichi-Ken, 480-1195, Japan |
| Dr. Yukihiro Horie | Obihiro Kyokai Hospital, Obihiro-shi, Hokkaido, 080-0805, Japan |
| Dr. Yuzo Suda | Dokkyo Medical University Hospital, Shimotsuga-gun, Tochigi-Ken, 321-0293, Japan |
| **Korea (South Korea)** | |
| Dr. Hakyoung Kim | Hallym University Kangnam Sacred Heart Hospital, Seoul, 150-950, Korea (Republic of Korea) |
| Dr. Hum Chung | Seoul National University Hospital, Seoul, Gyeonggi-do, 110744, Korea (Republic of Korea) |
| Prof. Hyoung Jun Koh | Gangnam Severance Hospital, Yonsei University Health System, Seoul, 062 73, Korea (Republic of Korea) |
| Dr. Hyun Woong Kim | Inje University Busan Paik Hospital, Busan, 47392, Korea (Republic of Korea) |
| Dr. Hyung-Woo Kwak | Kyung Hee University Hospital, Seoul, 024 47, Korea (Republic of Korea) |
| Dr. In Taek Kim | Kyungpook National University Hospital, Daegu, Gyeongsangbuk-do, 700-721, Korea (Republic of Korea) |
| Dr. Ji Eun Lee | Pusan National University Hospital, Busan, 602-739, Korea (Republic of Korea) |
| Dr. Ji Hun Song | Ajou University Hospital, Suwon-si, Gyeonggi-do, 16499, Korea (Republic of Korea) |
| Dr. Jong Woo Kim | Kim's Eye Hospital, Seoul, 150-034, Korea (Republic of Korea) |
| Dr. June-Gone Kim | Asan Medical Center, Seoul, 055 05, Korea (Republic of Korea) |
| Dr. Kyu Hyung Park | Seoul National University Bundang Hospital, Seongnam-si, Gyeonggi-do, 13620, Korea (Republic of Korea) |
| Dr. SeWoong Kang | Samsung Medical Center, Seoul, 063 51, Korea (Republic of Korea) |
| Dr. Won Ki Lee | The Catholic University of Korea, Seoul St. Mary’s Hospital, Seoul, 137-701, Korea (Republic of Korea) |
| Dr. Woohyok Chang | Yeungnam University Hospital, Daegu, 42415, Korea (Republic of Korea) |
| **Malaysia** | |
| Dr. Barkeh Hanim Bt Jumaat | International Specialist Eye Centre, Kuala Lumpur, Kuala Lumpur, 59200, Malaysia |
| Dr. Mae-Lynn Catherine Bastion | Pusat Perubatan Universiti Kebangsaan Malaysia, Kuala Lumpur, Kuala Lumpur, 56000, Malaysia |
| Dr. Nor Fariza Ngah | Hospital Selayang, Batu Caves, Selangor, 68100, Malaysia |
| Prof. Visvaraja Subrayan | University of Malaya Eye Research Centre, Kuala Lumpur, Kuala Lumpur, 50603, Malaysia |
| **Mexico** | |
| Dr. Abel Ramirez Estudillo | Hospital Oftalmológico Nuestra Señora de la Luz, Mexico City, Distrito Federal, 060 30, Mexico |
| Dr. Arturo Enriquez Huerta | Clínica de Ojos Monterrey S.A. de C.V., Monterrey, Nuevo León, 64060, Mexico |
| Dr. Ermilo Sanchez Buenfil | RetimediQ Centro de Retina y Oftalmologia Especializada, Merida, Yucatán, 97130, Mexico |
| Dr. Jesús González Cortez | Hospital Universitario Dr Jose E Gonzalez, Monterrey, Nuevo León, 64460, Mexico |
| Dr. Jose Dalma Weiszhausz | Dr. Alejandro Dalma y asoc., Mexico City, Distrito Federal, 11000, Mexico |
| Dr. Patricio Rodriguez Valdez | CIIES, Monterrey, Nuevo León, 64710, Mexico |
| Dr. René Cano Hidalgo | Instituto de Oftalmología Fundación Conde de la Valenciana, Mexico City, Distrito Federal, 068 00, Mexico |
| **Netherlands** | |
| Dr. Anne-Minke de Boer | OMC Amsterdam, Amsterdam, 1071NT, Netherlands |
| Dr. Janneke van Lith-Verhoeven | Sint Elisabeth Ziekenhuis Afd. Oogheelkunde, Tilburg, 5022 GC, Netherlands |
| Dr. Jenny Onkosuwito | Flevoziekenhuis, Almere, 1315 RA, Netherlands |
| **Panamá** | |
| Dr. Ana Paz | Clínica de Vitreo y Macula Dra. Ana Paz, Panamá, Panamá, Panamá |
| Dr. Roberto Yee | Clinica Yee, Panamá, Panamá, Panamá |
| **Peru** | |
| Dr. Giovanna Gonzalez Luey | Hospital Nacional Guillermo Almenara Irigoyen, Lima, 4559, Peru |
| Dr. Karen Barraza Lino | Instituto Oftalmosalud S.R.L, San Isidro, 15036, Peru |
| Dr. Miguel Guzman | Ophtalmology-TG Laser Oftalmica , Lima, 666, Peru |
| Dr. Silvio Lujan Najar | Macula D&T, San Isidro, 15036, Peru |
| **Poland** | |
| Dr. Ewa Fluder | Szpital Specjalistyczny im Sokołowskiego, Walbrzych, 58-300, Poland |
| Dr. Halina Wykrota | NZOZ Lens-Med, Katowice, 40-064, Poland |
| Dr. Ilona Pawlicka | Wojewódzki Szpital Okulistyczny w Krakowie, Krakow, 31-723, Poland |
| Dr. Jan Kucharczuk | 10 Wojskowy Szpital Kliniczny, Bydgoszcz, 85-681, Poland |
| Dr. Janusz Cieslik | NZOZ Medilens, Kielce, 25-514, Poland |
| Dr. Jerzy Mackiewicz | Medical University of Lublin, Lublin, 20-079, Poland |
| Prof. Krystyna Raczynska | Specjalistyczny Cabinet Lekarski Krystyna Raczynska, Gdansk, 80-147, Poland |
| Dr. Maciej Gawęcki | Szpital Specjalistyczny IM J.K. Lukowicza, Chojnice, 89-600, Poland |
| Dr. Maciej Gwóźdź | Samodzielny Publiczny ZOZ, Wolomin, 05-200, Poland |
| Dr. Małgorzata Figurska | Wojskowy Instytut Medyczny, Warszawa, 04-141, Poland |
| Mikołaj Meller | NZOZ Ocu Service, Poznan, 60-538, Poland |
| Dr. Piotr Gozdek | Szpital Zakonu Bonifratrów im. Św. Jana Bożego w Łodzi, Łódź, 93-357, Poland |
| Dr. Piotr Oleksy | Centrum Medyczne Uno-Med (Private Practice), Kalisz, 62-800, Poland |
| Dr. Sławomir Zalewski | Centrum Diagnostyki i Mikrochirurgii Oka LENS, Olsztyn, 10-424, Poland |
| **Portugal** | |
| Dr. Carla Teixeira | Hospital Pedro Hispano, Matosinhos, 4454-509, Portugal |
| Dr. João Chibante | Centro Hospitalar de Entre o Douro e Vouga, E.P.E - Hospital de São Sebastião, Santa Maria da Feira, 4520-211, Portugal |
| Dr. João Paulo Castro de Sousa | Centro Hospitalar Leiria - Hospital Santo André, Leiria, 2410-197, Portugal |
| Prof. José Cunha-Vaz | AIBILI, Coimbra, 3000-548, Portugal |
| Dr. Manuel Mariano | Centro Hospitalar do Baixo Vouga, E.P.E. – Unidade de Aveiro, Aveiro, 3814-501, Portugal |
| Dr. Rita Flores | HPP - Hospital dos Lusíadas, Lisbon, 1500-458, Portugal |
| Dr. Rufino Silva | Espaço Médico de Coimbra, Coimbra, 3030-163, Portugal |
| **Russia** | |
| Dr. Andrey Kuznetsov | MBHI City Clinical Hospital #11, Chelyabinsk, 454000, Russia |
| Prof. Andrey Shchuko | The Irkutsk Affiliate of Federal State Budgetary Institution "MNTK ye Microsurgery Complex" n.a. S.N, Irkutsk, 664033, Russia |
| Dr. Ekaterina Zakharova | Institution of Republic Sakha (Yakutiya) Yakutsk Republican Ophthalmology Hospital, Yakutsk, 677000, Russia |
| Dr. Elena Sumarokova | SBEI HPE "Saratov State Medical University n.a. V. I. Razumovskiy" of the MoH of the RF, Saratov, 410028, Russia |
| Dr. Elina Santoro | BI of Khanty-Mansyisk region Yugra "Surgut regional clinical hospital", Surgut, 628400, Russia |
| Dr. Elmira Abdulaeva | SAIH "Republican clinical ophthalmological hospital of MoH of Rebublic of Tatarstan", Kazan, 420012, Russia |
| Prof. Evgeniy Sergeevich Miludin | SBEI HPE "Samara State Medical University" of the MoH of the RF, Samara, 443099, Russia |
| Dr. Fedor Yevgenyevitch Shadrichev | Territorial Diabetic Center, Saint Petersburg, 194354, Russia |
| Dr. Galina Bratko | The S.N.Fyodorov Federal State Institution Eye Microsurgery Complex (Novosibirsk), Novosibirsk, 630071, Russia |
| Dr. Irina Mikhaylovna Rybina | Tyumen Regional Ophthalmology Dispensary, Tyumen, 625048, Russia |
| Dr. Lyubov Petrovna Danilova | The S.N.Fyodorov Federal State Institution Eye Microsurgery Complex (Khabarovsk), Khabarovsk, 680033, Russia |
| Dr. Maria Viktorovna Budzinskaya | Scientific Research Institute of Eye Diseases, Moscow, 119021, Russia |
| Dr. Marina Zimina | Chita State Medical Academy, Chita, 672090, Russia |
| Dr. Mukhtarram Mukhammarovitch Bikbov | SBI "Ufa scientific research institute of eye diseases of academy of sciences of the republic of Bas, Ufa, 450077, Russia |
| Dr. Nikolai Petrovitch Pashtaev | The S.N.Fyodorov Federal State Institution Eye Microsurgery Complex (Cheboxary), Cheboksary, 428028, Russia |
| Dr. Oleg L'vovich Fabrikantov | The S.N.Fyodorov Federal State Institution Eye Microsurgery Complex (Tambov), Tambov, 392000, Russia |
| Dr. Olga Vladimirovna Sarygina | Moscow Helmholtz Research Institute of Ophthalmology, Moscow, 105062, Russia |
| Dr. Sergey Sachnov | Krasnodar Branch of The S.N. Fyodorov FSBI "Eye microsurgery complex", Krasnodar, 350012, Russia |
| Dr. Timur Galeev | SBHI "Penza Regional Ophtalmological Hospital", Penza, 440026, Russia |
| Dr. Vitaly Sokolov | Dignostic Center №7, Saint Petersburg, 190000, Russia |
| **Saudi Arabia** | |
| Dr. Ammar Dawalibi | Prince Sultan Military Medical City, Riyadh, 11159, Saudi Arabia |
| Dr. Hassan Dhibi | King Khaled Eyes Specialist Hospital, Riyadh, 11462, Saudi Arabia |
| Dr. Karim Talaat | King Khalid National Guard Hospital, Jeddah, 21423, Saudi Arabia |
| Dr. Osama Alem | King Fahad National Guard Hospital, Riyadh, 14611, Saudi Arabia |
| **Singapore** | |
| Dr. Adrian Koh | Camden Medical Center, Singapore, 248649, Singapore |
| Dr. Gemmy Cheung | Singapore National Eye Centre, Singapore, 168751, Singapore |
| Prof. Tien Yin Wong | National University Hospital, Singapore, 119074, Singapore |
| **Slovakia** | |
| Dr. Alena Zencarova | Fakultna nemocnica s poliklinikou Nove Zamky, Nové Zámky, 940 01, Slovakia |
| Dr. Blandina Lipkova | Fakultna nemocnica s poliklinikou Zilina, Zilina, 010 01, Slovakia |
| Dr. Hedviga Miková | Nemocnica svateho Michala, Bratislava, 833 31, Slovakia |
| Dr. Jaroslav Hasa | Univerzitna nemocnica Bratislava, Nemocnica Ruzinov, Bratislava, 82606, Slovakia |
| Dr. Juraj Bajacek | Ustredna vojenska nemocnica SNP Ruzomberok- Fakultna nemocnica, Ruzomberok, 034 26, Slovakia |
| Dr. Livia Javorska | Nemocnica Poprad a.s., Poprad, 058 01, Slovakia |
| Dr. Maria Hurcikova | Nemocnica s poliklinikou Trebisov a.s., Trebišov, 075 01, Slovakia |
| Dr. Marta Ondrejkova | NsP Banská Bystrica, Banská Bystrica, 974 01, Slovakia |
| Dr. Monika Gajdosova | Oftal s.r.o., Zvolen, 960 01, Slovakia |
| Dr. Zuzana Jamrichova | Univerzitna nemocnica Bratislava, Nemocnica sv. Cyrila a Metoda, Bratislava, 851 07, Slovakia |
| Dr. Zuzana Sustykevicova | Fakultna nemocnica Trencin, Trenčín, 91101, Slovakia |
| **Slovenia** | |
| Dr. Ivana Gardaševič | General Hospital Novo Mesto, Novo Mesto, 8000, Slovenia |
| Dr. Marko Vrhovec | General Hospital Celje, Celje, 3000, Slovenia |
| Dr. Petra Skitek | University Medical Centre Maribor, Maribor, 2000, Slovenia |
| Dr. Polona Mekjavic | Univerzitetni Klinicni Center Ljubljana Ocesna Klinika, Ljubljana, 1000, Slovenia |
| **Spain** | |
| Dr. Belen Aurora Fente Sampayo | Hospital Universitario Lucus Augusti, Lugo, 27003, Spain |
| Dr. Emiliano Hernandez Galilea | Hospital Universitario de Salamanca, Salamanca, 37007, Spain |
| Dr. Enrique Lopez-Sanchez | Hospital Arnau de Vilanova, Valencia, 46015, Spain |
| Dr. Francisco Hurtado Cena | Clinica Rementeria, Madrid, 28010, Spain |
| Dr. Isabel Pinilla | Hospital Clinico Universitario Lozano Blesa, Zaragoza, 50009, Spain |
| Dr. Jesus Garcia Martinez | Hospital Moncloa, Madrid, 28008, Spain |
| Dr. Jose Buil Calvo | Hospital de la Santa Creu i Sant Pau, Barcelona, 080 25, Spain |
| Dr. Jose Escobar Barranco | Hospital Dos de Maig, Barcelona, 080 25, Spain |
| Dr. Juan Donate | Hospital Universitario Clinico San Carlos, Madrid, 28040, Spain |
| Dr. Laura Sararols | Fundacio Privada Hospital Asil de Granollers, Granollers, Barcelona, 084 02, Spain |
| Dr. Laura Sararols Ramsay | Hospital General de Catalunya, Sant Cugat del Valles, Barcelona, 8195, Spain |
| Dr. Lorenzo Lopez Guajardo | Hospital Universitario Principe de Asturias, Alcala de Henares, Madrid, 28805, Spain |
| Dr. Luis Moreno | Hospital Universitario 12 de Octubre, Madrid, 28041, Spain |
| Dr. Luis Pablo Julvez | Hospital Universitario Miguel Servet, Zaragoza, 50009, Spain |
| Dr. Manuel Franco Benito | Hospital Universitario de Leon, León, 24008, Spain |
| Dr. Manuel Gurrea | Centro Oftalmologico Gaztambide, Madrid, 28015, Spain |
| Dr. Manuela Contreras | Hospital Regional Universitario de Malaga, Málaga, 29010, Spain |
| Dr. Maribel Lopez-Galvez | Universidad de Valladolid, Valladolid, 47011, Spain |
| Dr. Marta Figueroa | Vissum Corporación Oftalmológica – Mirassierra, Madrid, 28035, Spain |
| Dr. Nuria Maria Gajate Paniagua | Hospital Universitario de Burgos, Burgos, 090 05, Spain |
| Dr. Pedro Aroca | Hospital Universitario Sant Joan de Reus, Reus, Tarragona, 431204, Spain |
| Dr. Roberto Gallego-Pinazo | Hospital Universitari i Politecnic La Fe, Valencia, 46026, Spain |
| Dr. Santiago Abengoechea | Centro de Oftalmología Barraquer, Barcelona, 8017, Spain |
| Dr. Sara Velilla Oses | Hospital San Pedro, Logrono, La Rioja, 26006, Spain |
| **Turkey** | |
| Prof. Berati Hasanreisoğlu | Gazi University Hospital, Ankara, 0 6560, Turkey |
| Prof. Bora Eldem | Hacettepe University Medical Faculty, Ankara, 0 6100, Turkey |
| Prof. Emin Ozmert | Ankara University Medical Faculty, Ankara, 0 6100, Turkey |
| Prof. Faruk Ozturk | Ankara Ataturk Training and Research Hospital, Ankara, 0 6100, Turkey |
| Dr. Rifat Rasier | Bilim University Florence Nightingale Hospital, Istanbul, 34387, Turkey |
| **Ukraine** | |
| Dr. Andriy Korol | V.P.Filatov Institute of Eye Diseases and Tissue Therapy AMS, Odesa, 65000, Ukraine |
| Dr. Andriy Petrunya | LLC “Lugansk Regional Central Eye Hospital”, Lugansk, 91055, Ukraine |
| Dr. Pavel Bezditko | Kharkov Regional Clinical Hospital, Kharkiv, 61022, Ukraine |
| Dr. Svjatoslav Suk | City clinical ophthalmological hospital, Kiev, 3065, Ukraine |
| Dr. Valerij Serdyuk | Regional clinical hospital n.a. Mechnikova, Dnipropetrovsk, 49027, Ukraine |
| **United Kingdom** | |
| Dr. Adam Booth | Queen Alexandra Hospital, Portsmouth, Hampshire, PO6 3LY, United Kingdom |
| Mr. Adam Ross | Bristol Eye Hospital (Retinal Treatment and Research Unit), Bristol, Avon, BS1 2LX, United Kingdom |
| Dr. Ahmed Kamal | University Hospital Aintree, NHS, Liverpool, Merseyside, L9 7AL, United Kingdom |
| Mr. Aires Lobo | Bedford Hospital,, Bedford, Bedfordshire, MK42 9DJ, United Kingdom |
| Prof. Andrew Lotery | Southampton General Hospital, Southampton, Hampshire, SO16 6YD, United Kingdom |
| Dr. Ashish Patwardhan | New Cross Hospital, Wolverhampton, West Midlands, WV10 0QP, United Kingdom |
| Dr. Benjamin Burton | James Paget University Hospital, Gorleston, Norfolk, NR31 6LA, United Kingdom |
| Mr. Bobby Paul | Queen's Hospital, Romford, Greater London, RM7 0AG, United Kingdom |
| Mr. Christopher Brand | Royal Hallamshire Hospital, Sheffield, South Yorkshire , S10 2JF, United Kingdom |
| Dr. Deepali Varma | Sunderland Eye Infirmary, Sunderland, Tyne & Wear, SR2 9HP, United Kingdom |
| Dr. Faruque Ghanchi | St Luke s Hospital Macula Clinic, Bradford, West Yorkshire, BD9 6RJ, United Kingdom |
| Ms. Geeta Menon | Frimley Park Hospital, Frimley, Surrey, GU16 7UJ, United Kingdom |
| Dr. Ian Pearce | Royal Liverpool University Hospital (Dept. St. Paul's Clinical Eye Research Centre), Liverpool, Norfolk, L7 8XP, United Kingdom |
| Mr. Javeed Khan | St Mary's Hospital, Newport, Isle of Wight, PO30 5TG, United Kingdom |
| Mr. Jignesh Patel | Essex County Hospital, Colchester, Essex, CO3 3NB, United Kingdom |
| Prof. Jonathan Gibson | Heart of England NHS Foundation Trust, Birmingham, West Midlands, B9 5SS, United Kingdom |
| Ms. Louise Downey | Hull and East Yorkshire Eye Hospital, Hull, North Yorkshire, HU32JZ, United Kingdom |
| Mr. Luke Membrey | Maidstone Hospital, Maidstone, Kent, ME16 9QQ, United Kingdom |
| Mr. Martin McKibbin | St James's University Hospital, Leeds, West Yorkshire, LS9 7TF, United Kingdom |
| Mr. Mohammed Musadiq | University Hospital of North Staffordshire Royal Infirmary, Stoke-on-Trent, Staffordshire, ST4 7LN, United Kingdom |
| Mr. Narendra Dhingra | Pinderfields Hospital GH, Wakefield, North Yorkshire, WF1 2DG, United Kingdom |
| Mr. Niral Karia | Southend University Hospital, Essex, SS0 0RY, United Kingdom |
| Mr. Nishal Patel | Kent and Canterbury Hospital, Kent, CT1 3NG, United Kingdom |
| Mr. Pankaj Puri | Royal Derby Hospital, Derby, Derbyshire, DE22 3NE, United Kingdom |
| Mr. Riaz Asaria | Royal Free Hospital, London, Greater London, NW3 2PF, United Kingdom |
| Mr. Richard Gale | York Hospital, York, North Yorkshire, YO31 8HE, United Kingdom |
| Mr. Rob Johnston | Cheltenham General Hospital, Cheltenham, Gloucestershire, GL54 7AN, United Kingdom |
| Mr. Robin Hamilton | Moorfields Eye Hospital, London, Greater London, EC1V 2PD, United Kingdom |
| Mr. Saad Younis | Western Eye Hospital, London, Greater London, NW1 5QH, United Kingdom |
| Mr. Sajjad Haider | University Hospital North Durham, Durham, DH1 5TW, United Kingdom |
| Mr. Sajjad Mahmood | Manchester Royal Eye Hospital, Manchester, Greater Manchester, M13 9WL, United Kingdom |
| Mr. Salman Mirza | Worcestershire Royal Hospital, Worcester, Worcestershire, WR5 1DD, United Kingdom |
| Mrs. Salwa Abugreen | Royal Blackburn Infirmary, Blackburn, Lancashire, BB2 3LR, United Kingdom |
| Mr. Samer El-Sherbiny | Birmingham and Midland Eye Centre, Birmingham, West Midlands, United Kingdom |
| Mr. Sanjiv Banerjee | University Hospital of Wales, Cardiff, CF14 4XW, United Kingdom |
| Mr. Sergio Pagliarini | Hospital St Cross Macular Unit, Rugby, West Midlands, CV225PH, United Kingdom |
| Ms. Sheena George | Hillingdon Hospital NHS Foundation Trust, Uxbridge, Greater London, UB8 3NN, United Kingdom |
| Dr. Simon Hewick | Raigmore Hospital, Inverness, Highland Region, IV2 3UJ, United Kingdom |
| Mr. Simon Kelly | Royal Bolton Hospital, Bolton, Lancashire, BL4 0JR, United Kingdom |
| Dr. Simon Taylor | Royal Surrey County Hospital, Guildford, Surrey, GU2 7XX, United Kingdom |
| Ms. Sohba Sivaprasad | King s College Hospital, London, Greater London, SE5 9RS, United Kingdom |
| Mr. Somnath Banerjee | Leicester Royal Infirmary, Leicester, Leicestershire, LE1 5WW, United Kingdom |
| Dr. Sudeshna Patra | Whipps Cross University Hospital, London, Greater London, E11 1NR, United Kingdom |
| Ms. Susan Downes | John Radcliffe Hospital, Oxford, Oxfordshire, OX3 7RP, United Kingdom |
| Dr. Tanya Moutray | Royal Victoria Hospital, Belfast, BT12 6 BA, United Kingdom |
| Mr. Tarek El-Kashab | Leighton Hospital, Crewe, Cheshire, CW1 4QJ, United Kingdom |
| Mr. Vineeth Kumar | Arrowe Park Hospital, Upton, Merseyside, CH49 5PE, United Kingdom |
| Mrs. Yinka Osoba | Torbay Hospital, Torquay, Devon, TQ2 7AA, United Kingdom |
| Dr. Yit Yang | New Cross Hospital, Wolverhampton, West Midlands, WV10 0QP, United Kingdom |
