# Supplementary material for: Effectiveness and safety of ranibizumab 0.5 mg in treatment-naïve patients with diabetic macular edema: Results from the real-world global LUMINOUS study
Source: PLoS One. 2020 Jun 3;15(6):e0233595. doi: 10.1371/journal.pone.0233595 (PMC7269267; doi:10.1371/journal.pone.0233595)
Supplement: S2 Appendix — (PDF) [file pone.0233595.s002.pdf]

List of Independent Ethics Committees (IECs) or Institutional Review Boards (IRBs).

List of Independent Ethics Committees (IEC) or Institutional Review Boards (IRB) by study center

Center Number	Ethics Committee or Institutional Review Board	Department / Organization	EC/IRB City	EC/IRB State/ Province	EC/IRB Postal Code	Center Country
1100	Comite Independiente de Etica para Ensayos en Famacologia Clinica	Consultorio de Investigaciones Oftalmológicas	Buenos Aires	Buenos Aires	C1027AAP	Argentina
1101	Comité de Ética en Investigación Instituto de Investigaciones Clínicas	Clínica Privada de Ojos José León Suárez	Buenos Aires	Buenos Aires	C1027AAP	Argentina
1102	Comite Independiente de Etica para Ensayos en Famacologia Clinica	Consultorio Dr. Andres Jakofsky	Buenos Aires	Buenos Aires	C1027AAP	Argentina
1103	Comite Independiente de Etica para Ensayos en Famacologia Clinica	Clínica de Ojos	Buenos Aires	Entre Rios	C1027AAP	Argentina
1104	Comite Independiente de Etica para Ensayos en Famacologia Clinica	DYTER S.A.	Buenos Aires	Mendoza	C1027AAP	Argentina
1105	Comite Independiente de Etica para Ensayos en Famacologia Clinica	Clínica de Ojos Srl	Buenos Aires	Santa Fe	C1027AAP	Argentina
1106	Comite Independiente de Etica para Ensayos en Famacologia Clinica	Microcirugia Ocular (Clínica MICRO)	Buenos Aires	Santa Fe	C1027AAP	Argentina
1107	Comite Independiente de Etica para Ensayos en Famacologia Clinica	Instituto Donato	Buenos Aires	Ciudad Autonoma Buenos Aires	C1027AAP	Argentina
1108	Comite Independiente de Etica para Ensayos en Famacologia Clinica	Instituto de la Visión	Buenos Aires	Ciudad Autonoma Buenos Aires	C1027AAP	Argentina
1109	Comité de Ética en Investigación Instituto de Investigaciones Clínicas	*Oftalmologia Integral*	Mar del Plata	Buenos Aires	B7600FZN	Argentina
1110	Comité de Ética en Investigación Instituto de Investigaciones Clínicas	Clinica Modelo de Lanus	Buenos Aires	Buenos Aires	C1027AAP	Argentina
1111	Comite Independiente de Etica para Ensayos en Famacologia Clinica	Centro Oftalmologico Ventola	Buenos Aires	Buenos Aires	C1027AAP	Argentina
1113	Comite Independiente de Etica para Ensayos en Famacologia Clinica	Clínica Dr Rivero Covre	Buenos Aires	Santa Fe	C1027AAP	Argentina
1114	Comite Independiente de Etica Fundacion Rusculleda	Instituto Oftamológico de Córdoba SA	Cordoba	Cordoba	X5003DC E	Argentina
1115	Comite de Etica de CER Investigaciones Clinicas (CECIC)	Hospital Oftalmológico Malvinas Argentinas	Quilmes	Buenos Aires	B1878DVB	Argentina
1116	Comite Independiente de Etica para Ensayos en Famacologia Clinica	Consultorios Oftalmologico Dres Fugazzotto	Buenos Aires	Mendoza	C1027AAP	Argentina
1117	Comite Independiente de Etica para Ensayos en Famacologia Clinica	Plaza Vision S.A.	Buenos Aires	Mendoza	C1027AAP	Argentina

1118	Comite Independiente de Etica para Ensayos en Famacologia Clinica	Grupo laser Visión - Rosario Eximer Laser Visión	Buenos Aires	Santa Fe	C1027AAP	Argentina
1119	Comite de Etica de CER Investigaciones Clinicas (CECIC)	Clínica Oftalmológica Meroni	Quilmes	Buenos Aires	B1878DVB	Argentina
3001	Ethik-Kommision Der Medizinischen Universitat Wien	General University Hospital of Vienna	Wien	Vienna	1090	Austria
3002	Ethik-Kommision Der Medizinischen Universitat Wien	Prof. Dr. Siegfried Priglinger	Wien	Vienna	1090	Austria
2000	NSW Government Health Sydney Local Health District	Sydney Eye Hospital, Research Development Office	Camperdo wn	New South Wales	2050	Australia
2001	Bellberry Human Research Ethics Committee	Lions Eye Institute	North Adelaide	South Australia	5065	Australia
2002	Human Research Ethics Committee (Tasmania) Network	Hobart Eye Surgeions, University of Tasmania	Hobart	Tasmania	7001	Australia
2003	Bellberry Human Research Ethics Committee	Eye Consultants SA	North Adelaide	South Australia	5065	Australia
2004	Bellberry Human Research Ethics Committee	Vision Eye Institute Chatswood	North Adelaide	South Australia	5065	Australia
2005	Tasmania Health and Medical Human Research Ethics Committee	Office of Research Services, University of Tasmania	Hobart	Tasmania	7001	Australia
2006	Bellberry Human Research Ethics Committee	Retina & Vitreous Centre	North Adelaide	New South Wales	5065	Australia
2007	Bellberry Human Research Ethics Committee	Vision Retinal Institute	North Adelaide	Queenslan d	5065	Australia
2008	Bellberry Human Research Ethics Committee	Forster Eye Surgery	North Adelaide	New South Wales	5065	Australia
2010	The Royal Victorian Eye & Ear Hospital	Human Research & Ethics Committee	East Melbourne	Victoria	8002	Australia
2011	Bellberry Human Research Ethics Committee	Brisbane Eye Clinic	North Adelaide	Queenslan d	5065	Australia
2012	Bellberry Human Research Ethics Committee	Sydney Retina Eye Clinic and Day Surgery	North Adelaide	New South Wales	5065	Australia
2013	Bellberry Human Research Ethics Committee	Macquarie University	North Adelaide	New South Wales	5064	Australia
2014	Bellberry Human Research Ethics Committee	Marsden Eye Specialists	North Adelaide	New South Wales	5065	Australia
2017	Bellberry Human Research Ethics Committee	Southern Ophthalmology	North Adelaide	New South Wales	5065	Australia
2018	Bellberry Human Research Ethics Committee	Strathfield Retina Clinic	North Adelaide	New South Wales	5065	Australia
2019	Bellberry Human Research Ethics Committee	Retina Associates - Chatswood Retina Service	North Adelaide	New South Wales	5065	Australia
2020	Bellberry Human Research Ethics Committee	Macquarie University	North Adelaide	New South Wales	5065	Australia
2021	Bellberry Human Research Ethics Committee	Adelaide Eye and Retina Centre	North Adelaide	South Australia	5065	Australia
2024	Bellberry Human Research Ethics Committee	Queensland Eye Institute	North Adelaide	Queenslan d	5065	Australia

2025	Bellberry Human Research Ethics Committee	Melbourne Retina Associates	North Adelaide	Victoria	5065	Australia
2026	Bellberry Human Research Ethics Committee	Private Rooms, Eye Clinic (B4a) Westmead Hospital	North Adelaide	New South Wales	5065	Australia
2032	Bellberry Human Research Ethics Committee	St. John of God Hospital	North Adelaide	Victoria	5065	Australia
2034	Bellberry Human Research Ethics Committee	Oakleigh Eye Center	North Adelaide	Victoria	5065	Australia
2035	Bellberry Human Research Ethics Committee	Retina and Vitreous Centre	North Adelaide	New South Wales	5065	Australia
2037	Bellberry Human Research Ethics Committee	Waverley Eye Clinic	North Adelaide	Victoria	5065	Australia
3100	Committee for Medical Ethics/Clinical Research	Faculty of Medicine UZ Gathuisberg	Leuven		3000	Belgium
3101	Committee for Medical Ethics/Clinical Research	Faculty of Medicine UZ Gathuisberg	Leuven	Hasselt	3000	Belgium
3102	Committee for Medical Ethics/Clinical Research	Faculty of Medicine UZ Gathuisberg	Leuven		3000	Belgium
3104	Committee for Medical Ethics/Clinical Research	Faculty of Medicine UZ Gathuisberg	Leuven		3000	Belgium
3106	Committee for Medical Ethics/Clinical Research	Faculty of Medicine UZ Gathuisberg	Leuven		3000	Belgium
3107	Committee for Medical Ethics/Clinical Research	Faculty of Medicine UZ Gathuisberg	Leuven		3000	Belgium
3110	Committee for Medical Ethics/Clinical Research	Faculty of Medicine UZ Gathuisberg	Leuven		3000	Belgium
3111	Committee for Medical Ethics/Clinical Research	Faculty of Medicine UZ Gathuisberg	Leuven		3000	Belgium
3112	Committee for Medical Ethics/Clinical Research	Faculty of Medicine UZ Gathuisberg	Leuven		3000	Belgium
3113	Comite d'Ethique Centre Hospitalier de Mouscron	Centre Hospitalier de Mouscron	Mouscron		7700	Belgium
3114	Committee for Medical Ethics/Clinical Research	Faculty of Medicine UZ Gathuisberg	Leuven		3000	Belgium
3117	Committee for Medical Ethics/Clinical Research	Faculty of Medicine UZ Gathuisberg	Leuven		3000	Belgium
3118	Committee for Medical Ethics/Clinical Research	Faculty of Medicine UZ Gathuisberg	Leuven		3000	Belgium
3120	Committee for Medical Ethics/Clinical Research	Faculty of Medicine UZ Gathuisberg	Leuven		3000	Belgium
1200	Hospital Municipal Dr. Mario Gatti	Centro Medico de Oftalmologia	Cambui	Campinas	13092	Brazil
1203	Comissao Nacional de Etica Em Pesquisa	Instituto da Visão	Belo Horizonte	Minas Gerais	30150	Brazil
1204	Comissao Nacional de Etica Em Pesquisa	HFSE - Hospital Federal dos Servidores do Estado do Rio de Janeiro	Belo Horizonte	Rio de Janeiro	30150	Brazil
1205	Comissao Nacional de Etica Em Pesquisa	CBCO - Centro Brasileiro de Cirurgia de Olhos	Belo Horizonte	Goiás	30150	Brazil
1208	Comissao Nacional de Etica Em Pesquisa	ANGIOCORPORE	Belo Horizonte	Sao Paulo	30150	Brazil
1209	Comissao Nacional de Etica Em Pesquisa	Clínica Lavinsky Oftalmologia	Belo Horizonte	Rio Grande do Sul	30150	Brazil
1000	Ontario IRB/REB	IRB Services	Aurora	New Brunswick	L4G 0A5	Canada

1001	Ontario IRB/REB	IRB Services	Aurora	Nova Scotia	L4G 0A5	Canada
1003	Ontario IRB/REB	IRB Services	Aurora	Ontario	L4G 0A5	Canada
1008	Ontario IRB/REB	IRB Services	Aurora	Ontario	L4G 0A5	Canada
1007	University Health Network	Research Ethics Board	Toronto	Ontario	M5G 1Z5	Canada
1006	Ontario IRB/REB	IRB Services	Aurora	Ontario	L4G 0A5	Canada
1005	Ontario IRB/REB	IRB Services	Aurora	Ontario	L4G 0A5	Canada
1004	Ontario IRB/REB	IRB Services	Aurora	Quebec	L4G 0A5	Canada
1010	Ontario IRB/REB	IRB Services	Aurora	Quebec	L4G 0A5	Canada
1011	Ottawa Health Science Network Research Ethics Board	The Ottawa Hospital - General Campus, University of Ottawa Eye Institute	Ottawa	Ontario	K1Y 4E9	Canada
1012	Ontario IRB/REB	The Retina Centre of Ottawa	Aurora	Ontario	L4G 0A5	Canada
1013	Ontario IRB/REB	IRB Services	Aurora	Ontario	L4G 0A5	Canada
1014	Ontario IRB/REB	IRB Services	Aurora	British Columbia	L4G 0A5	Canada
1015	Health Research Ethics Board of Alberta	IRB Services	Edmonton	Alberta	T5J 4A7	Canada
1016	Ontario IRB/REB	Clincial Trials Committee	Aurora	Ontario	L4G 0A5	Canada
1018	Ontario IRB/REB	IRB Services	Aurora	Ontario	L4G 0A5	Canada
1020	Ontario IRB/REB	IRB Services	Aurora	Ontario	L4G 0A5	Canada
1021	Ontario IRB/REB	IRB Services	Aurora	Ontario	L4G 0A5	Canada
1019	Ontario IRB/REB	IRB Services	Aurora	Ontario	L4G 0A5	Canada
8200	Resolucion Comite Etico Cientifico	Hospital del Cobre Salvador Allende Gossens	La Serena		1399001	Chile
2101	Beijing Tongren Hospital EC	Renmin Hospital of Wuhan University	Wuhan	Hubei	430060	China
2102	Beijing Tongren Hospital EC	No. 10 People's Hospital of Shanghai		Shanghai	200072	China
2104	Beijing Tongren Hospital EC	Eye and ENT hospital		Shanghai	200000	China
2106	Beijing Tongren Hospital EC	Zhongshan Ophthalmic Center, Sun Yat-sen University	Guangzhou	Guangdong	510060	China
2107	Beijing Tongren Hospital EC	Peking University First Hospital	Beijing,P. R.	Beijing	100034	China
2108	Beijing Tongren Hospital EC	Tianjin Medical University Eye Center	Tianjin		300384	China
2109	Beijing Tongren Hospital EC	Xinhua Hospital Affiliated to Shanghai Jiao Tong University School of Medicine		Shanghai	200092	China
2100	Beijing Tongren Hospital EC	Beijing Tong Ren Hospital, Capital Medical University		Beijing	100730	China
2115	Beijing Tongren Hospital EC	Peking University Third Hospital		Beijing	100191	China
2116	Beijing Tongren Hospital EC	Chinese PLA General Hospital	Beijing	Beijing	100853	China
2117	Beijing Tongren Hospital EC	Beijing Hospital		Beijing	100730	China
2119	Beijing Tongren Hospital EC	Peking Union Medical College Hospital		Beijing	100032	China
2121	Beijing Tongren Hospital EC	Southwest Hospital	Chongqing	Chongqing	400038	China
2122	Beijing Tongren Hospital EC	Shanghai First People's Hospital		Shangai	200080	China
2124	Beijing Tongren Hospital EC	Xiamen Eye Centre	Fujian		361001	China

2127	Beijing Tongren Hospital EC	Shierming Eye Hospital	Jinan	Shandong	250001	China
2128	Beijing Tongren Hospital EC	The Affiliated Hospital of Guiyang Medical College	Guiyang	Guizhou	500000	China
2126	Beijing Tongren Hospital EC	No. 474 Hospital of PLA	Wulumuqi	Xinjiang Uygur	830000	China
2130	Beijing Tongren Hospital EC	No.2 Hospital Affiliated to Jilin University	Changchu n City	Jilin	130041	China
2129	Beijing Tongren Hospital EC	Yunnan 2nd People's Hospital	Kunming	Yun'nan	650021	China
1304	Sociedad de Cirugia Ocular S.A.	Sociedad de Cirugía Ocular	Bogota			Colombia
1302	Instituto para Ninos Ciegos y Sordos del Valle del Cauca	Instituto de Ciegos y Sordos INSORP	San Fernando	Cali		Colombia
1306	Sociedad de Cirugia Ocular S.A.	Clínica Barraquer	Bogota			Colombia
1307	Sociedad de Cirugia Ocular S.A.	Clinica Oftalmologica	Bogota			Colombia
1309	Comite de Etica en la Investigacion	OPTISALUD SAS	Bogota			Colombia
1308	Sociedad de Cirugia Ocular S.A.	Clínica Oftalmológica del Atlántico	Bogota			Colombia
3300	Eticka komise Fakultni nemocnice Hradec Kralove	Fakultni nemocnice Hradec Kralove	Hradec Kralove		500 05	Czech Republic
3302	Eticka komise Fakultni Nemocnice Kralovske Vinohrady	Fakultni nemocnice Kralovske Vinohrady	Praha		100 34	Czech Republic
3303	Eticka komise FN Ostrava	University Hopsital Ostrava	Ostrava-Poruba		70800	Czech Republic
3305	Eticka komise Vseobecne fakultni nemocnice v Praze	Charles University Hopsital 1st Faculty of Medicine	Praha 2	Praha 2	128 08	Czech Republic
3307	Eticka komise Fakultni Nemocnice Brno	Fakultni nemocnice Brno	Brno		625 00	Czech Republic
3306	Eticka komise Ustredni vojenske nemocnice	Ustredni vojenska nemocnice Praha	Praha 6		169 02	Czech Republic
3301	Eticka komise-Krajska zdravotni a.s.	Krajska zdravotni, a.s. - Masarykova nemocnice v Usti nad Labem	Usti nad Labem		40113	Czech Republic
3308	Eticka komise FN a LF UP Olomouc-LEC	Fakultni nemocnice Olomouc	Olomouc		775 20	Czech Republic
3304	Eticka komise Fakultni nemocnice Plzen	Fakultni nemocnice Plzen	Plzen		305 99	Czech Republic
3600	Ethikkommission an der Medizinischen Fakultaet der Rheinischen Friedrich-Wilhelms-Universitaet Bonn	University of Bonn	Bonn	Nordrhein Westfalen	53105	Germany
3601	Aerztekammer Berlin	Praxis Arzt für Augenheilkunde	Berlin		10969	Germany
3604	Ethik-Kommission der Ärztekammer Westfalen-Lippe und der Med. Fakultät der Universität Münster	St. Franziskus Hospital	Muenster	Nordrhein Westfalen	48147	Germany
3602	Ethikkommission an der Medizinischen Fakultaet der Rheinischen Friedrich-Wilhelms-Universitaet Bonn	Augenarztpraxis Grasbon	Bonn	Bayern	53105	Germany
3606	Aerztekammer Berlin	Praxis Steinberg	Berlin		10969	Germany
3607	Ethik-Kommission der Ärztekammer Westfalen-Lippe und der Med.	Universitaetsklinikum Muenster	Muenster	Nordrhein Westfalen	48147	Germany

	Fakultät der Universität Münster					
3608	Ethikkommission der erztekammer Hamburg	Praxis_Dr Kaupke	Hamburg		22083	Germany
3609	Ethik-Kommission der Ärztekammer Westfalen-Lippe und der Med. Fakultät der Universität Münster	Klinikum Lüdenscheid	Muenster	Nordrhein Westfalen	48147	Germany
3611	Landesaerztekammer Baden-Wuerttemberg	Dr. Rabethge Klinik GmbH	Stuttgart	Baden Wuerttem berg	70597	Germany
3612	Universitaetsklinikum Tuebingen	Eberhard Karls University Eye Hospital	Tuebingen	Baden Wuerttem berg	72074	Germany
3615	Ethik-Kommission der Aerztekammer Niedersachsen	Klinikum Osnabrück	Hannover	Niedersac hsen	30175	Germany
3616	Geschaeftsstelle der Ethikkommission	Universitaetsklinikum Koeln	Koeln	Nordrhein Westfalen	50931	Germany
3628	Augenlaserzentrum Neu-Ulm	Augenlaserzentrum	Neu-Ulm	Bayern	89231	Germany
3629	Ethik-Kommission der Aerztekammer Niedersachsen	Augenklinik Dannenberg	Hannover	Niedersac hsen	30175	Germany
3632	Ethik-Kommission der Ärztekammer Westfalen-Lippe und der Westfälischen Wilhelms-Universität Münster	Private practice_ Dr Grote-Schmidt	Münster	Nordrhein Westfalen	48147	Germany
3631	An die Ethik-Kommission der Bayerischen Landesärztekammer	Fachärztin für Augenheilkunde	Munich	Bayern	81677	Germany
3633	An die Ethik-Kommission der Bayerischen Landesärztekammer	Klinikum Augsburg Augenklinik	Munich	Bayern	81677	Germany
3630	An die Ethik-Kommission der Bayerischen Landesärztekammer	Dr. med. Claus Fuchs Fachärzte für Augenheilkunde	Munich	Bayern	81677	Germany
8300	Comite de Bioetica de la Universidad Central del Ecuador (COBI-UCE)	Centro Medico Quirurgico Oftalmologico Alta Vision	Quito		593	Ecuador
4600	CEIC Hospital Universitario Ramon y Cajal	Vissum Corporación Oftalmológica – Mirassierra	Madrid		28034	Spain
4601	CEIC Hospital Universitario Ramon y Cajal	Hospital Clinico Universitario Lozano Blesa	Madrid		28035	Spain
4602	CEIC Hospital Universitario Ramon y Cajal	Hospital Universitari i Politecnic La Fe	Madrid		28036	Spain
4604	CEIC Hospital Universitario Ramon y Cajal	Hospital Universitario Clinico San Carlos	Madrid		28039	Spain
4605	CEIC Hospital Universitario Ramon y Cajal	Hospital Universitario 12 de Octubre	Madrid		28040	Spain
4606	CEIC Hospital Universitario Ramon y Cajal	Hospital Universitario Miguel Servet	Madrid		28043	Spain
4608	CEIC Hospital Universitario Ramon y Cajal	Hospital de la Santa Creu i Sant Pau	Madrid		28045	Spain
4612	CEIC Hospital Universitario Ramon y Cajal	Universidad de Valladolid	Madrid		28049	Spain
4614	CEIC Hospital Universitario Ramon y Cajal	Hospital General de Catalunya	Madrid	Barcelona	28049	Spain

4615	CEIC Hospital Universitario Ramon y Cajal	Hospital San Pedro	Madrid	La Rioja	28049	Spain
4620	CEIC Hospital Universitario Ramon y Cajal	Hospital Universitario Principe de Asturias	Madrid	Madrid	28049	Spain
4622	CEIC Hospital Universitario Ramon y Cajal	Hospital Universitario de Burgos	Madrid		28049	Spain
4623	CEIC Hospital Universitario Ramon y Cajal	Hospital Universitario de Salamanca	Madrid		28049	Spain
4624	CEIC Hospital Universitario Ramon y Cajal	Hospital Moncloa	Madrid		28049	Spain
4625	CEIC Hospital Universitario Ramon y Cajal	Hospital Universitario de Leon	Madrid		28049	Spain
4626	CEIC Hospital Universitario Ramon y Cajal	Hospital Universitario Lucus Augusti	Madrid		28049	Spain
4627	CEIC Hospital Universitario Ramon y Cajal	Hospital Dos de Maig	Madrid	Barcelona	28050	Spain
4628	CEIC Hospital Universitario Ramon y Cajal	Hospital Arnau de Vilanova	Madrid		28050	Spain
4630	CEIC Hospital Universitario Ramon y Cajal	Clinica Rementeria	Madrid		28051	Spain
4634	CEIC Hospital Universitario Ramon y Cajal	Centro de Oftalmología Barraquer	Madrid		28052	Spain
4632	CEIC Hospital Universitario Ramon y Cajal	Hospital Regional Universitario de Malaga	Madrid		28053	Spain
4631	CEIC Hospital Universitario Ramon y Cajal	Hospital Universitario Sant Joan de Reus	Madrid	Tarragona	28054	Spain
4629	CEIC Hospital Universitario Ramon y Cajal	Fundacio Privada Hospital Asil de Granollers	Madrid	Barcelona	28055	Spain
4633	CEIC Hospital Universitario Ramon y Cajal	Centro Oftalmologico Gaztambide	Madrid		28056	Spain
3500	N/A	Centre Hospitalier Intercommunal de Créteil	Creteil	Val de Marne		France
3501	N/A	Centre Ophtalmologique d'Imagerie et de Laser	Paris	Val de Marne	94010	France
3502	N/A	Clinique Ocean	Vannes	Côte-d'Or	75015	France
3503	N/A	CHU Dijon - Hopital General	Dijon	Côte-d'Or	56000	France
3504	N/A	Visiopole Private Practice	Lagord	Charente	21000	France
3505	N/A	CHU de Nice - Hôpital Lenval	Nice	Alpes Maritimes	17140	France
3506	N/A	Cabinet Odeon	Paris		060 06	France
3507	N/A	Groupe Hospitalier Pellegrin -Hôpital Pellegrin	Bordeaux	Gironde	75006	France
3518	N/A	Centre Ophtalmologique de L'Odeon	Paris		33000	France
3508	N/A	Hôpital Lariboisière	Paris		75006	France
3509	N/A	Polyclinique de Courlancy	Reims	Marne	75475	France
3510	N/A	Clinique Orl Honore Cave	Montauban	Tarn et Garonne	51100	France
3511	N/A	Cabinet d'Ophtalmologie	Melun	Seine et Marne	82000	France

3512	N/A	CHU Nantes - Hôtel Dieu	Nantes Cedex 1	Loire Atlantique	77000	France
3513	N/A	Centre Hospitalier de la Croix Rousse	Lyon	Rhone	44093	France
3514	N/A	CHU Toulouse, Hôpital Paule de Vignier	Toulouse	Cedex 9	69317	France
3516	N/A	Fondation Ophtalmologique Adolphe de Rothschild	Paris		31059	France
3517	N/A	Clinique de Montargis	Montargis	Loiret	75019	France
5000	NRES Committee Yorkshire and the Humber Leeds East	Central Ethics Committee	Jarrow	Lancashir e	45200	United Kingdom
5001	NRES Committee Yorkshire and the Humber Leeds East	Central Ethics Committee	Jarrow	South Yorkshire	NE 32 3DT	United Kingdom
5002	NRES Committee Yorkshire and the Humber Leeds East	Central Ethics Committee	Jarrow	Tyne & Wear	NE 32 3DT	United Kingdom
5003	NRES Committee Yorkshire and the Humber Leeds East	Central Ethics Committee	Jarrow		NE 32 3DT	United Kingdom
5004	NRES Committee Yorkshire and the Humber Leeds East	Central Ethics Committee	Jarrow	Surrey	NE 32 3DT	United Kingdom
5005	NRES Committee Yorkshire and the Humber Leeds East	Central Ethics Committee	Jarrow	North Yorkshire	NE 32 3DT	United Kingdom
5006	NRES Committee Yorkshire and the Humber Leeds East	Central Ethics Committee	Jarrow	Greater Manchest er	NE 32 3DT	United Kingdom
5009	NRES Committee Yorkshire and the Humber Leeds East	Central Ethics Committee	Jarrow		NE 32 3DT	United Kingdom
5030	NRES Committee Yorkshire and the Humber Leeds East	Central Ethics Committee	Jarrow	Greater London	NE 32 3DT	United Kingdom
5010	NRES Committee Yorkshire and the Humber Leeds East	Central Ethics Committee	Jarrow	Norfolk	NE 32 3DT	United Kingdom
5011	NRES Committee Yorkshire and the Humber Leeds East	Central Ethics Committee	Jarrow	Staffordshi re	NE 32 3DT	United Kingdom
5012	NRES Committee Yorkshire and the Humber Leeds East	Central Ethics Committee	Jarrow		NE 32 3DT	United Kingdom
5013	NRES Committee Yorkshire and the Humber Leeds East	Central Ethics Committee	Jarrow	West Midlands	NE 32 3DT	United Kingdom
5014	NRES Committee Yorkshire and the Humber Leeds East	Central Ethics Committee	Jarrow	West Midlands	NE 32 3DT	United Kingdom
5015	NRES Committee Yorkshire and the Humber Leeds East	Central Ethics Committee	Jarrow	West Yorkshire	NE 32 3DT	United Kingdom
5024	NRES Committee Yorkshire and the Humber Leeds East	Central Ethics Committee	Jarrow	Surrey	NE 32 3DT	United Kingdom
5055	NRES Committee Yorkshire and the Humber Leeds East	Central Ethics Committee	Jarrow	Greater London	NE 32 3DT	United Kingdom
5036	NRES Committee Yorkshire and the Humber Leeds East	Central Ethics Committee	Jarrow	Gloucester shire	NE 32 3DT	United Kingdom
5037	NRES Committee Yorkshire and the Humber Leeds East	Central Ethics Committee	Jarrow	Greater London	NE 32 3DT	United Kingdom
5016	NRES Committee Yorkshire and the Humber Leeds East	Central Ethics Committee	Jarrow	West Midlands	NE 32 3DT	United Kingdom

5029	NRES Committee Yorkshire and the Humber Leeds East	Central Ethics Committee	Jarrow	Hampshire	NE 32 3DT	United Kingdom
5021	NRES Committee Yorkshire and the Humber Leeds East	Central Ethics Committee	Jarrow		NE 32 3DT	United Kingdom
5017	NRES Committee Yorkshire and the Humber Leeds East	Central Ethics Committee	Jarrow	Hampshire	NE 32 3DT	United Kingdom
5022	NRES Committee Yorkshire and the Humber Leeds East	Central Ethics Committee	Jarrow	Cheshire	NE 32 3DT	United Kingdom
5018	NRES Committee Yorkshire and the Humber Leeds East	Central Ethics Committee	Jarrow	Greater London	NE 32 3DT	United Kingdom
5019	NRES Committee Yorkshire and the Humber Leeds East	Central Ethics Committee	Jarrow	North Yorkshire	NE 32 3DT	United Kingdom
5020	NRES Committee Yorkshire and the Humber Leeds East	Central Ethics Committee	Jarrow	North Yorkshire	NE 32 3DT	United Kingdom
5026	NRES Committee Yorkshire and the Humber Leeds East	Central Ethics Committee	Jarrow	West Midlands	NE 32 3DT	United Kingdom
5025	NRES Committee Yorkshire and the Humber Leeds East	Central Ethics Committee	Jarrow	West Midlands	NE 32 3DT	United Kingdom
5044	NRES Committee Yorkshire and the Humber Leeds East	Central Ethics Committee	Jarrow	Leicestershire	NE 32 3DT	United Kingdom
5031	NRES Committee Yorkshire and the Humber Leeds East	Central Ethics Committee	Jarrow	Isle of Wight	NE 32 3DT	United Kingdom
5032	NRES Committee Yorkshire and the Humber Leeds East	Central Ethics Committee	Jarrow	Lancashire	NE 32 3DT	United Kingdom
5023	NRES Committee Yorkshire and the Humber Leeds East	Central Ethics Committee	Jarrow	West Yorkshire	NE 32 3DT	United Kingdom
5028	NRES Committee Yorkshire and the Humber Leeds East	Central Ethics Committee	Jarrow	Greater London	NE 32 3DT	United Kingdom
5033	NRES Committee Yorkshire and the Humber Leeds East	Central Ethics Committee	Jarrow	Greater London	NE 32 3DT	United Kingdom
5038	NRES Committee Yorkshire and the Humber Leeds East	Central Ethics Committee	Jarrow	Oxfordshire	NE 32 3DT	United Kingdom
5027	NRES Committee Yorkshire and the Humber Leeds East	Central Ethics Committee	Jarrow	Essex	NE 32 3DT	United Kingdom
5039	NRES Committee Yorkshire and the Humber Leeds East	Central Ethics Committee	Jarrow	Greater London	NE 32 3DT	United Kingdom
5034	NRES Committee Yorkshire and the Humber Leeds East	Central Ethics Committee	Jarrow	Merseyside	NE 32 3DT	United Kingdom
5040	NRES Committee Yorkshire and the Humber Leeds East	Central Ethics Committee	Jarrow	Devon	NE 32 3DT	United Kingdom
5041	NRES Committee Yorkshire and the Humber Leeds East	Central Ethics Committee	Jarrow	Highland Region	NE 32 3DT	United Kingdom
5042	NRES Committee Yorkshire and the Humber Leeds East	Central Ethics Committee	Jarrow		NE 32 3DT	United Kingdom
5047	NRES Committee Yorkshire and the Humber Leeds East	Central Ethics Committee	Jarrow	Norfolk	NE 32 3DT	United Kingdom
5035	NRES Committee Yorkshire and the Humber Leeds East	Central Ethics Committee	Jarrow	Avon	NE 32 3DT	United Kingdom

5043	NRES Committee Yorkshire and the Humber Leeds East	Central Ethics Committee	Jarrow	Kent	NE 32 3DT	United Kingdom
5046	NRES Committee Yorkshire and the Humber Leeds East	Central Ethics Committee	Jarrow	Derbyshire	NE 32 3DT	United Kingdom
5060	NRES Committee Yorkshire and the Humber Leeds East	Central Ethics Committee	Jarrow	Bedfordshire	NE 32 3DT	United Kingdom
5061	NRES Committee Yorkshire and the Humber Leeds East	Central Ethics Committee	Jarrow	Merseyside	NE 32 3DT	United Kingdom
5062	NRES Committee Yorkshire and the Humber Leeds East	Central Ethics Committee	Jarrow	Worcestershire	NE 32 3DT	United Kingdom
3700	Scientific Council of University Eye Hospital of Haraklion	University Eye Hospital of Heraklion	Heraklion		71201	Greece
3701	Scientific Council of Eye Hospital of Athens	Eye Hospital of Athens -1st Clinic	Athens		10672	Greece
3702	Scientific Council of Omma Opthalmological Institute of Athens	Omma Ophtalmological Institute of Athens	Athens		11525	Greece
3703	Scientific Council of General Hospital Papageorgiou	General Hospital Papageorgiou	Thessaloniki		56429	Greece
3704	Scientific Council of Red Cross Hospital	Red Cross Hospital - 2nd Clinic	Athens		11526	Greece
3705	Scientific Council of University Hospital of Alexandroupolis	University General Hospital of Alexandroupolis	Alexandroupolis		68100	Greece
3710	Ethics Committee of University Hospital of Larissa	University General Hospital of Larissa	Mezourlo		41110	Greece
3712	Scientific Council of Athens Vision Eye Institute	Athens Vision Eye Institute	Athens		17673	Greece
3714	Scientific Council of Athens Eye Hospital	Ophtalmiatrio Athinon - Athens Eye Hospital	Athens		10672	Greece
3715	Ethics Committee of General Hospital of Lamia	General Hospital of Lamia	Lamia		35100	Greece
3716	Ethics Committee of University Hospital of Patra	University Hopsital of Patras	Patra		26504	Greece
3717	Ethics Committee of General Hospital of Rethymnon	General Hospital of Rethymnon	Rethymnon		74100	Greece
8800	Research Ethics Committee (Kowloon Central/Kowloon East)	Hong Kong Eye Hospital	Kowloon		N/A	Hong Kong
3800	Egeszsegugyi Tudomanyos Tanacs Tudomanyos es Kutatasetikai Bizottsag	Budapest Retina Associates	Budapest		1051	Hungary
3802	Egeszsegugyi Tudomanyos Tanacs Tudomanyos es Kutatasetikai Bizottsag	Semmelweis Egyetem	Budapest		1051	Hungary
3801	Egeszsegugyi Tudomanyos Tanacs Tudomanyos es Kutatasetikai Bizottsag	Debreceni Egyetem Klinikai Kozpont	Budapest		1051	Hungary
3803	Egeszsegugyi Tudomanyos Tanacs Tudomanyos es Kutatasetikai Bizottsag	Pecsi Tudomanyegyetem	Budapest		1051	Hungary
3804	Egeszsegugyi Tudomanyos Tanacs Tudomanyos es Kutatasetikai Bizottsag	Szegedi Tudomanyegyetem Szent-Gyorgyi Albert Klinikai Kozpont	Budapest		1051	Hungary

3900	Beacon Hospital Research Ethics Committee	Beacon Clinic	Sandyford		18	Ireland
3901	Research Ethics Committee Southeast Area	Whitfield Clinic	Waterford		X91	Ireland
3902	Mater Misericordiae Univ Hospital Research Ethics Committee	Mater Private Hospital	Dublin	Dublin	7	Ireland
3903	Research Ethics Committee Southeast Area	Waterford Regional Hospital	Waterford		X91	Ireland
8400	Helsinki Committee, Rabin MC	Rabin Medical Center-Beilinson Campus	Petah Tikva		49100	Israel
8401	Helsinki Committee, Sourasky MC	Tel Aviv Sourasky Medical Center	Tel-Aviv		64239	Israel
8402	Helsinki Committee, Bnai-Zion	Bnai Zion Medical Center	Haifa		31048	Israel
6003	Prasad Eye Institute Ethics Committee	L. V. Prasad Eye Institute	Hyderabad	Andhra Pradesh	500034	India
6002	All India Institute of Medical Sciences Ethics Committee	All India Institute of Medical Sciences	Ansari Nagar	Delhi	110029	India
6000	Aravind Eye Care System Ethical Committee	Aravind Eye Hospital	Coimbator e	Tamilnadu	641014	India
6004	Sangini Hospital Ethics Committee	Bankers Eye Institute	Ahmedaba d	Gujarat	380006	India
6005	Sparsh Hospital Ethics Committee	L. V. Prasad Eye Institute	Bhubanes war	Orissa	751007	India
6008	Vision Research Foundation Ethics Sub-Committee	Sankara Nethralaya	Chennai	Tamilnadu	600006	India
6010	National Institute of Ophthalmology Ethics Committee	National Institute of Ophthalmology	Pune	Maharasht ra	411005	India
4001	Comitato Etico Interaziendale	Reparto diOculistica dell Ospedale SS Antonio e Biagio e Cesare Arrigo de Alessandria	Alessandri a		15121	Italy
4002	Comitato Etico Indipendente - Azienda Ospedaliero-Univeritaria	Ente Ecclesiastico Ospedale Generale Regionale F Miulli	Bari	Bari	70124	Italy
4003	Reggio Calabria Comitato Etico	Azienda Ospedaliera Bianchi Melacrino Morelli	Reggio Calabria		89100	Italy
4004	Comitato Etico Dell 'Universita' Cattolica Del Sacro Cuore	Policlinico Universitario Agostino Gemelli	Roma		001 68	Italy
4005	Spedali Civili - Brescia Comitato Etico Provinciale	Azienda Socio Sanitaria Territoriale degli Spedali Civili di Brescia (Presidio Spedali Civili)	Brescia		25123	Italy
4006	Comitato per la Sperimentazione Clinica Medicinali dell'Az Ospedaliero Universitaria Pisana d. Pisa	Azienda Ospedaliero Universitaria Cisanello	Pisa		56126	Italy
4008	Regione Autonoma Della Sardegna Azienda Ospedaliero Universitaria Di Cagliari Comitato Etico Indipendente	Ospedale S.Giovanni di Dio	Cagliari		091 24	Italy
4009	Comitato Etico Dell 'Universita' Dell' Universita' "Sapienza"	Umberto I Pol. di Roma-Universita di Roma La Sapienza	Roma		001 61	Italy
4010	Regione del Veneto Aziendo U.L.S.S. n. 1	Ospedale San Martino di Belluno	Belluno		27717	Italy

6154	Fukushima Medical University Hospital Institutional Review Board	Fukushima Medical University Hospital	Fukushima-shi	Fukushima-Ken	960-1295	Japan
6155	Sapporo City General Hospital The Ethical Committee of Sapporo City General Hospital	Sapporo City General Hospital	Sapporo-shi	Hokkaido	060-8604	Japan
6115	Asahikawa Medical University Hospital Institutional Review Board	Asahikawa Medical University Hospital	Asahikawa-shi	Hokkaido	078-8510	Japan
6161	Tohoku University Hospital Institutional Review Board	Tohoku University Hospital	Sendai-shi	Miyagi-Ken	980-8574	Japan
6116	Akita University Hospital Institutional Review Board	Akita University Hospital	Akita-shi	Akita-Ken	010-8543	Japan
6100	Hattori Clinic Institutional Review Board	Shuhokai Ohtsuka Eye Hospital	Sapporo-shi	Hokkaido	001-0016	Japan
6175	Aomori Prefectural Central Hospital the ethical committee in Aomori Prefectural Central Hospital	Aomori Prefectural Central Hospital	Aomori	Aomori	030-8553	Japan
6117	Kitami Red Cross Hospital Institutional Review Board	Kitami Red Cross Hospital	Kitami-shi	Hokkaido	090-8666	Japan
6118	Jusendo General Hospital Independent Ethics Committee	Jusendo General Hospital	Koriyama-shi	Fukushima-Ken	963-8585	Japan
6182	Yamagata University Hospital Institutional Review Board	Yamagata University Hospital	Yamagata-shi	Yamagata-Ken	990-9585	Japan
6101	Hattori Clinic Institutional Review Board	Yoshida Eye Hospital	Hakodate-shi	Hokkaido	041-0851	Japan
6119	Gunma University Hospital Institutional Review Board	Gunma University Hospital	Maebashi-shi	Gunma-Ken	371-8511	Japan
6107	Tokyo Medical University Ibaraki Medical Center Institutional Review Board	Tokyo Medical University Ibaraki Medical Center	Inashiki-gun	Ibaraki-Ken	300-0395	Japan
6102	Hattori Clinic Institutional Review Board	Aoyagi Eye Clinic	Ueda-shi	Nagano-Ken	386-0002	Japan
6120	Dokkyo Medical University Hospital Institutional Review Board	Dokkyo Medical University Hospital	Shimotsuga-gun	Tochigi-Ken	321-0293	Japan
6121	Jichi Medical University Hospital Institutional Review Board	Jichi Medical University Hospital	Shimotsuke-shi	Tochigi-Ken	329-0498	Japan
6103	Matsumoto Dental University Hospital Institutional Review Board	Matsumoto Dental University Hospital	Shiojiri-shi	Nagano-Ken	399-0781	Japan
6122	Tsukuba University Hospital Institutional Review Board	Tsukuba University Hospital	Tsukuba-shi	Ibaraki-Ken	305-8576	Japan
6162	Saitama Medical University Hospital Institutional Review Board	Saitama Medical University Hospital	Iruma-gun	Saitama-Ken	350-0495	Japan
6163	Toho University Sakura Medical Center Independent Ethics Committee	Toho University Sakura Medical Center	Sakura-shi	Chiba-Ken	285-8741	Japan
6199	Juntendo University Urayasu Hospital Institutional Review Board	Juntendo University Urayasu Hospital	Urayasu-shi	Chiba-Ken	279-0021	Japan
6123	Saitama Red Cross Hospital Institutional Review Board	Saitama Red Cross Hospital	Saitama-shi	Saitama-Ken	338-8553	Japan
6156	Hattori Clinic Institutional Review Board	Hoeikai Hoshiai Eye Clinic	Saitama-shi	Saitama-Ken	336-0963	Japan
6104	Yokohama City University Medical Center Institutional Review Board	Yokohama City University Medical Center	Yokohama-shi	Kanagawa-Ken	232-0024	Japan

6124	Yokohama City University Hospital Institutional Review Board	Yokohama City University Hospital	Yokohama -shi	Kanagawa -Ken	236-0004	Japan
6125	Yokosuka Kyosai Hospital Institutional Review Board	Yokosuka Kyosai Hospital	Yokohama -shi	Kanagawa -Ken	236-0004	Japan
6183	Seirei Hamamatsu General Hospital Institutional Review Board	Seirei Hamamatsu General Hospital	Hamamats u-shi	Shizuoka-Ken	430-8558	Japan
6108	St. Marianna University School of Medicine Hospital Institutional Review Board	St. Marianna University School of Medicine Hospital	Kawasaki-shi	Kanagawa -Ken	216-8511	Japan
6126	Shizuoka Saiseikai General Hospital Internal Review Board	Shizuoka Saiseikai General Hospital	Shizuoka-shi	Shizuoka-Ken	422-8527	Japan
6127	Juntendo University Shizuoka Hospital Institutional Review Board	Juntendo University Shizuoka Hospital	Izunokuni-shi	Shizuoka-Ken	410-2295	Japan
6109	Hattori Clinic Institutional Review Board	Ishikawa Eye Clinic	Shizuoka-shi	Shizuoka-Ken	420-0841	Japan
6176	Hattori Clinic Institutional Review Board	Kaiya Ophthalmology	Hamamats u-shi	Shizuoka-Ken	430-0903	Japan
6105	Hattori Clinic Institutional Review Board	Totsukaekimae Suzuki Eye Clinic	Yokohama -shi	Kanagawa -Ken	244-0003	Japan
6177	Nihon University Hospital Institutional Review Board	Nihon University Hospital	Chiyoda-ku	Tokyo-To	101-8309	Japan
6164	Kyorin University Hospital Institutional Review Board	Kyorin University Hospital	Mitaka-shi	Tokyo-To	181-8611	Japan
6165	Tokyo Medical University Hospital Institutional Review Board	Tokyo Medical University Hospital	Shinjuku-ku	Tokyo-To	160-0023	Japan
6128	University of Yamanashi Hospital Institutional Review Board	University of Yamanashi Hospital	Chuo-shi	Yamanash i-Ken	409-3898	Japan
6129	Saiando Ochanomizu Inoue Eye Clinic Institutional Review Board	Saiando Ochanomizu Inoue Eye Clinic	Chiyoda-ku	Tokyo-To	101-0062	Japan
6130	Tokyo Medical University Hachioji Medical Center Institutional Review Board	Tokyo Medical University Hachioji Medical Center	Hachioji-shi	Tokyo-To	193-0998	Japan
6187	Juntendo University Hospital Institutional Review Board	Juntendo University Hospital	Bunkyo-ku	Tokyo-To	113-8431	Japan
6173	Tokyo Women's Medical University Hospital Institutional Review Board	Tokyo Women's Medical University Hospital	Shinjuku-ku	Tokyo-To	162-8666	Japan
6131	Nagoya University Hospital Institutional Review Board	Nagoya University Hospital	Nagoya-shi	Aichi-Ken	466-8560	Japan
6132	Mie University Hospital Internal Review Board	Mie University Hospital	Tsu-shi	Mie-Ken	514-8507	Japan
6133	Hattori Clinic Institutional Review Board	Shozankai Miyake Eye Hospital	Nagoya-shi	Aichi-Ken	462-0825	Japan
6134	JCHO Chukyo Hospital Institutional Review Board	JCHO Chukyo Hospital	Nagoya-shi	Aichi-Ken	457-8510	Japan
6188	Fujita Health University Hospital Institutional Review Board	Fujita Health University Hospital	Toyoake-shi	Aichi-Ken	470-1192	Japan
6110	Hattori Clinic Institutional Review Board	Koyokai Yayoi Hospital	Toyohashi -shi	Aichi-Ken	441-8106	Japan
6178	Aichi Medical University Hospital Institutional Review Board	Aichi Medical University Hospital	Nagakute-shi	Aichi-Ken	480-1195	Japan
6136	Hattori Clinic Institutional Review Board	Nishijima Eye Clinic	Kyoto-shi	Kyoto-Fu	604-0837	Japan
6157	Shiga University of Medical Science Hospital Institutional Review Board	Shiga University of Medical Science Hospital	Otsu-shi	Shiga-Ken	520-2192	Japan

6189	Kyoto University Hospital Institutional Review Board	Kyoto University Hospital	Kyoto-shi	Kyoto-Fu	606-8507	Japan
6158	Kanazawa University Hospital Institutional Review Board	Kanazawa University Hospital	Kanazawa -shi	Ishikawa-Ken	920-8641	Japan
6190	Toyama University Hospital Institutional Review Board	Toyama University Hospital	Toyama-shi	Toyama-Ken	930-0194	Japan
6166	Osaka University Hospital Institutional Review Board	Osaka University Hospital	Suita-shi	Osaka-Fu	565-0871	Japan
6179	Kansai Medical University Hospital Institutional Review Board	Kansai Medical University Hospital	Hirakata-shi	Osaka-Fu	573-1191	Japan
6191	Hyogo College of Medicine Hospital Institutional Review Board	Hyogo College of Medicine Hospital	Nishinomiya-shi	Hyogo-Ken	663-8501	Japan
6200	Hyogo Prefectural Amagasaki General Medical Center Institutional Review Board	Hyogo Prefectural Amagasaki General Medical Center	Amagasaki-shi	Hyogo-Ken	660-8550	Japan
6137	Kansai Medical University Medical Center Institutional Review Board	Kansai Medical University Takii Hospital	Moriguchi-shi	Osaka-Fu	570-8507	Japan
6138	Steel Memorial Hirohata Hospital Institutional Review Board	Steel Memorial Hirohata Hospital	Himeji-shi	Hyogo-Ken	671-1122	Japan
6159	Nara Medical University Hospital Institutional Review Board	Nara Medical University Hospital	Kashihara-shi	Nara-Ken	634-8522	Japan
6184	Kobe Kaisei Hospital Institutional Review Board	Kobe Kaisei Hospital	Kobe-shi	Hyogo-Ken	657-0068	Japan
6160	Kindai University Hospital Independent Ethics Committee	Kindai University Hospital	Osakasayama-shi	Osaka-Fu	589-8511	Japan
6202	Japan Red Cross Society Wakayama Medical Center Independent Ethics Committee	Japan Red Cross Society Wakayama Medical Center	Wakayama-shi	Wakayama-Ken	640-8558	Japan
6180	Kakogawa City West Hospital Institutional Review Board	Kakogawa City West Hospital	Kakogawa -shi	Hyogo-Ken	675-8611	Japan
6139	Kagawa University Hospital Institutional Review Board	Kagawa University Hospital	Kita-gun	Kagawa-Ken	761-0793	Japan
6140	Hiroshima University Hospital Institutional Review Board	Hiroshima University Hospital	Hiroshima-shi	Hiroshima-Ken	734-8551	Japan
6141	Ehime University Hospital Independent Ethics Committee	Ehime University Hospital	Toon-shi	Ehime-Ken	791-0295	Japan
6185	Tokushima University Hospital Institutional Review Board	Tokushima University Hospital	Tokushima -shi	Tokushima-Ken	770-8503	Japan
6167	Kochi Medical School Hospital Institutional Review Board	Kochi Medical School Hospital	Nankoku-shi	Kochi-Ken	783-8505	Japan
6111	Matsuyama Red Cross Hospital Institutional Review Board	Matsuyama Red Cross Hospital	Matsuyama-shi	Ehime-Ken	790-8524	Japan
6192	Kyushu University Hospital Institutional Review Board	Kyushu University Hospital	Higashi-ku	Fukuoka	812-8582	Japan
6143	Japanese Red Cross Nagasaki Genbaku Hospital Independent Ethics Committee	Japanese Red Cross Nagasaki Genbaku Hospital	Nagasaki-shi	Nagasaki-Ken	852-8511	Japan
6144	Kagoshima University Hospital Institutional Review Board	Kagoshima University Medical And Dental Hospital	Kagoshima-shi	Kagoshima-Ken	890-8520	Japan
6193	University of Miyazaki Hospital Institutional Review Board	University of Miyazaki Hospital	Miyazaki-shi	Miyazaki-Ken	889-1692	Japan

6168	Meiwakai Miyata Ophthalmic Hospital Institutional Review Board	Meiwakai Miyata Ophthalmic Hospital	Miyakonojo-shi	Miyazaki-Ken	885-0051	Japan
6194	Fukuoka University Hospital Institutional Review Board	Fukuoka University Hospital	Fukuoka-shi	Fukuoka-Ken	814-0180	Japan
6145	Nagasaki University Hospital Institutional Review Board	Nagasaki University Hospital	Nagasaki-shi	Nagasaki-Ken	852-8501	Japan
6195	University of Occupational and Environmental Health Hospital Internal Review Board	University of Occupational and Environmental Health Hospital	Kitakyushu-shi	Fukuoka-Ken	807-8556	Japan
6169	Meiwakai Kagoshima Miyata Ophthalmic Hospital Institutional Review Board	Meiwakai Kagoshima Miyata Ophthalmic Hospital	Kagoshima-shi	Kagoshima-Ken	890-0046	Japan
6106	Hattori Clinic Institutional Review Board	Taidokai Sato Ganka Iin Domachi Clinic	Yamagata-shi	Yamagata-Ken	990-0051	Japan
6146	Yonezawa City Hospital Institutional Review Board	Yonezawa City Hospital	Yonezawa-shi	Yamagata-Ken	992-8502	Japan
6170	JOHAS Tohoku Rosai Hospital Institutional Review Board	JOHAS Tohoku Rosai Hospital	Sendai-shi	Miyagi-Ken	981-8563	Japan
6114	Meiji University of Integrative Medicine Institutional Review Board	Meiji University of Integrative Medicine	Nantan-shi	Kyoto-Fu	629-0392	Japan
6174	Obihiro Kyokai Hospital Institutional Review Board	Obihiro Kyokai Hospital	Obihiro-shi	Hokkaido	080-0805	Japan
6171	Sapporo Medical University Hospital Institutional Review Board	Sapporo Medical University Hospital	Sapporo-shi	Hokkaido	060-8543	Japan
6148	Ogaki Tokushukai Hospital Tokushu-kai Ethics Committee	Ogaki Tokushukai Hospital	Ogaki-shi	Gifu-Ken	503-0015	Japan
6112	Hattori Clinic Institutional Review Board	Tagawa Eye Clinic	Kanazawa-shi	Ishikawa-Ken	920-1151	Japan
6186	University of Fukui Hospital Institutional Review Board	University of Fukui Hospital	Yoshida-gun	Fukui-Ken	910-1193	Japan
6150	Japanese Red Cross Society Suwa Hospital Institutional Review Board	Japanese Red Cross Society Suwa Hospital	Suwa-shi	Nagano-Ken	392-8510	Japan
6151	Hattori Clinic Institutional Review Board	Hirota Eye Clinic	Shunan-shi	Yamaguchi-Ken	745-0017	Japan
6113	Hattori Clinic Institutional Review Board	Dannoue Eye Clinic	Kawasaki-shi	Kanagawa-Ken	211-0053	Japan
6152	Hattori Clinic Institutional Review Board	Jigankai Sanjo Eye Clinic	Sanjo-shi	Niigata-Ken	955-0852	Japan
6153	Hattori Clinic Institutional Review Board	Infinity Medical Group Kondo Eye Clinic	Hachioji-shi	Tokyo-To	192-0081	Japan
6172	Hattori Clinic Institutional Review Board	Musashi Dream Eye Clinic	Osaka-shi	Osaka-Fu	543-0027	Japan
6181	Toho University Ohashi Medical Center Institutional Review Board	Toho University Ohashi Medical Center	Meguro-ku	Tokyo-To	153-8515	Japan
6196	Hattori Clinic Institutional Review Board	Shinseikai Toyama Hospital	Imizu-shi	Toyama-Ken	939-0243	Japan
6197	Hattori Clinic Institutional Review Board	Ando Eye Clinic	Ashigarakami-gun	Kanagawa-Ken	258-0003	Japan
6198	Juntendo University Nerima Hospital Institutional Review Board	Juntendo University Nerima Hospital	Nerima-ku	Tokyo-To	177-8521	Japan
6600	Kangnam Sacred Heart Hospital IRB Membership List	Hallym University Kangnam Sacred Heart Hospital	Seoul		150-950	Korea, Republic of
6601	Seoul National University Bundang Hospital	Seoul National University Bundang Hospital	Seongnam-si	Gyeonggi-do	13620	Korea, Republic of

6602	Seoul National University Hospital Institutional Review Board	Seoul National University Hospital	Seoul	Gyeonggi-do	110744	Korea, Republic of
6603	Kim's Eye Hospital IRB	Kim's Eye Hospital	Seoul		150-034	Korea, Republic of
6604	Seoul St. Mary's Hospital, The Catholic University of Korea IRB	The Catholic University of Korea, Seoul St. Mary’s Hospital	Seocho-gu		137-701	Korea, Republic of
6605	The Institutional Review Board of Kyungpook National University Hospital	Kyungpook National University Hospital	Daegu	Gyeongsangbuk-do	700-721	Korea, Republic of
6606	Yonsei University Gangnam Severance Hospital, Institutional Review Board	Gangnam Severance Hospital, Yonsei University Health System	Gangnam-gu		062 73	Korea, Republic of
6607	PNUH Institutional Review Board	Pusan National University Hospital	Busan		602-739	Korea, Republic of
6608	IRB of Inje University Busan Paik Hospital	Inje University Busan Paik Hospital	Busan		47392	Korea, Republic of
6609	Yeungnam University Hospital Institutional Review Board	Yeungnam University Hospital	Daegu		42415	Korea, Republic of
6610	Asan Medical Center IRB	Asan Medical Center	Seoul		055 05	Korea, Republic of
6612	KHUH IRB	Kyung Hee University Hospital	Seoul		024 47	Korea, Republic of
6652	Samsung Medical Center Institutional Review Board	Samsung Medical Center	Seoul		063 51	Korea, Republic of
6611	The Institutional Review Board of Ajou University Hospital	Ajou University Hospital	Gyeonggi-do	Gyeonggi-do	16499	Korea, Republic of
1400	Comite de Etica en Investigacion de la facultad de Medicina y Hospital Universitario de la Universidad Autonoma de Nuevo Leon	Hospital Universitario Dr Jose E Gonzalez	Monterrey	Nuevo León	64460	Mexico
1401	Comite de Etica en Investigacion de la Escuela de Medicina del Instituto Tecnologico y de Estudios Superiores de Monterrey	CIIES	Monterrey	Nuevo León	64710	Mexico
1403	Comite de Investigacion Instituo de Oftalmologia Fundacion de Asistencia Privada	Instituto de Oftalmología Fundación Conde de la Valenciana	Mexico City	Distrito Federal	0 6800	Mexico
1405	Comite de Etica e Investigacion Fundacion Hospital "Nuestra Senora de la Luz"	Hospital Oftalmológico Nuestra Señora de la Luz	Mexico City	Distrito Federal	0 6030	Mexico
1406	Comite de Investigaction de la Clinica Bajio CLINBA S.C.	Clínica de Ojos Monterrey S.A. de C.V.	Guanajuato	Nuevo León	36090	Mexico
1412	Comite Independiente de Etica de Investigación y Bioseguridad del Bajio SC	Dr. Alejandro Dalma y asoc.	Guanajuato	Distrito Federal	36090	Mexico
1413	Comite Independiente de Etica de Investigación y Bioseguridad del Bajio SC	RetimediQ Centro de Retina y Oftalmologia Especializada	Guanajuato	Yucatán	36090	Mexico
5100	Medical Ethics Committee, University Malaya Medical Centre	University of Malaya Eye Research Centre	Kuala Lumpur	Kuala Lumpur	59100	Malaysia
5101	Medical Research Ethics Committee, Ministry of Health Malaysia	Hospital Selayang	Kuala Lumpur	Selangor	59000	Malaysia

5102	Medical Research & Ethics Committee Kementerian Kesihatan Malaysia	International Specialist Eye Centre	Kuala Lumpur	Kuala Lumpur	59000	Malaysia
5103	Universiti Kebangsaan Malaysia Medical Centre	Pusat Perubatan Universiti Kebangsaan Malaysia	Kuala Lumpur	Kuala Lumpur	56000	Malaysia
4100	Medisch Ethische Toetsingscommissie	Sint Elisabeth Ziekenhuis Afd. Oogheelkunde	Tilburg	Noord-Brabant	5022 GC	Netherlands
4101	Medisch Ethische Toetsingscommissie	OMC Amsterdam	Tilburg	Noord-Brabant	5022 GC	Netherlands
4103	Medisch Ethische Toetsingscommissie	Flevoziekenhuis	Tilburg	Noord-Brabant	5022 GC	Netherlands
8500	Hospital Nacional Guillermo Almenara Irigoyen Comite de Etica en Investigacion	Hospital Nacional Guillermo Almenara Irigoyen	Lima		15036	Peru
8501	Comite Institucional de Etica en Investigacion de la Universidad de San Martin de Porres	Instituto Oftalmosalud S.R.L	Lima		15036	Peru
8502	Comite Institucional de Etica en Investigacion de la Universidad de San Martin de Porres	Macula D&T	Lima		15036	Peru
8503	Prisma ONG	Ophtalmology-TG Laser Oftalmica	Lima		15036	Peru
4200	Ethics Committee of Silesian Medical Chamber	Samodzielny Publiczny ZOZ	Grazynskiego		40-126	Poland
4205	Ethics Committee of Silesian Medical Chamber	NZOZ Lens-Med	Grazynskiego		40-126	Poland
4201	Ethics Committee of Silesian Medical Chamber	10 Wojskowy Szpital Kliniczny	Grazynskiego		40-126	Poland
4206	Ethics Committee of Silesian Medical Chamber	Szpital Specjalistyczny im Sokołowskiego	Grazynskiego		40-126	Poland
4202	Ethics Committee of Silesian Medical Chamber	NZOZ Ocu Service	Grazynskiego		40-126	Poland
4203	Ethics Committee of Silesian Medical Chamber	Specjalistyczny Cabinet Lekarski Krystyna Raczynska	Grazynskiego		40-126	Poland
4204	Ethics Committee of Silesian Medical Chamber	NZOZ Medilens	Grazynskiego		40-126	Poland
4211	Ethics Committee of Silesian Medical Chamber	Wojewódzki Szpital Okulistyczny w Krakowie	Grazynskiego		40-126	Poland
4212	Ethics Committee of Silesian Medical Chamber	Centrum Diagnostyki i Mikrochirurgii Oka LENS	Grazynskiego		40-126	Poland
4213	Ethics Committee of Silesian Medical Chamber	Szpital Specjalistyczny IM J.K. Lukowicza	Grazynskiego		40-126	Poland
4217	Ethics Committee of Silesian Medical Chamber	Centrum Medyczne Uno-Med (Private Practice)	Grazynskiego		40-126	Poland
4219	Ethics Committee of Silesian Medical Chamber	Szpital Zakonu Bonifratrów im. Św. Jana Bożego w Łodzi	Grazynskiego		40-126	Poland
4220	Ethics Committee of Silesian Medical Chamber	Wojskowy Instytut Medyczny	Grazynskiego		40-126	Poland
4221	Ethics Committee of Silesian Medical Chamber	Medical University of Lublin	Grazynskiego		40-126	Poland
4300	Aibili Comissao de Etica para a Saude	AIBILI	Porto Salvo		2740-262	Portugal
4303	Hospital Lusiadas Lisboa Comissao de Etica para a	HPP - Hospital dos Lusíadas	Lisbon		1500-458	Portugal

	Saude					
4304	Centro Hospitalar de Entre o Douro e Vouga, E.P.E.	Centro Hospitalar de Entre o Douro e Vouga, E.P.E - Hospital de São Sebastião	Santa Maria da Feira		4520-211	Portugal
4306	Centro Hospitalar Leiria Comissao de Etica	Centro Hospitalar Leiria - Hospital Santo André	Leira		2410-197	Portugal
4301	Centro Hospitalar Leiria Comissao de Etica	Hospital Pedro Hispano	Leira		2410-197	Portugal
4302	Centro Hospitalar Do Baixo Vouga, E.P.E./Aveiro	Centro Hospitalar do Baixo Vouga, E.P.E. – Unidade de Aveiro	Aveiro		3814-501	Portugal
4305	CEIC - Parque de Saude de Lisboa	Espaço Médico de Coimbra	Lisboa		1749-004	Portugal
4407	The Committee of Biomedical Ethics of Ufa Institute of scientific-research of eye diseases of Academy of Sciences (Bashkortostan)	SBI "Ufa scientific research institute of eye diseases of academy of sciences of the republic of Bas	Ufa		450077	Russia
4400	The Ethics Committee of "The Postgraduating Doctors' Training Institute" of the Healthcare and Social Development Ministry of the Chuvash Republic	The S.N.Fyodorov Federal State Institution Eye Microsurgery Complex (Cheboxary)	Cheboksar y		428003	Russia
4412	The Ethics Committee of GBUZ SOCOB n.a. T.I. Eroshevsky	SBEI HPE "Samara State Medical University" of the MoH of the RF			443099	Russia
4423	The Independent Multidisciplinary Committee on Ethical Review of Clinical Trials	The Irkutsk Affiliate of Federal State Budgetary Institution "MNTK ye Microsurgery Complex" n.a. S.N	Moscow		125468	Russia
4403	The Independent Multidisciplinary Committee on Ethical Review of Clinical Trials	Institution of Republic Sakha (Yakutiya) Yakutsk Republican Ophthalmology Hospital	Moscow		125468	Russia
4402	The Independent Multidisciplinary Committee on Ethical Review of Clinical Trials	The S.N.Fyodorov Federal State Institution Eye Microsurgery Complex (Khabarovsk)	Moscow		125468	Russia
4404	The Independent Multidisciplinary Committee on Ethical Review of Clinical Trials	Territorial Diabetic Center	Moscow		125468	Russia
4401	The Independent Multidisciplinary Committee on Ethical Review of Clinical Trials	Dignostic Center №7	Moscow		125468	Russia
4406	The Ethics Committee of "Federal State budgetary Institution "Scientific Research Institute of Eye Diseases" of Russian Academy of medical Sciences	Scientific Research Institute of Eye Diseases	Moscow		119021	Russia
4408	The Ethics Committee of The Helmholtz Moscow Research Institute of Eye Diseases	Moscow Helmholtz Research Institute of Ophthalmology	Moscow		105062	Russia

4405	The Independent Multidisciplinary Committee on Ethical Review of Clinical Trials	Tyumen Regional Ophthalmology Dispensary	Moscow		125468	Russia
4419	The Independent Multidisciplinary Committee on Ethical Review of Clinical Trials	Chita State Medical Academy	Moscow		125468	Russia
4418	The Independent Multidisciplinary Committee on Ethical Review of Clinical Trials	The S.N.Fyodorov Federal State Institution Eye Microsurgery Complex (Tambov)	Moscow		125468	Russia
4420	The Independent Multidisciplinary Committee on Ethical Review of Clinical Trials	SBHI "Penza Regional Ophtalmological Hospital"	Moscow		125468	Russia
4421	The Independent Multidisciplinary Committee on Ethical Review of Clinical Trials	BI of Khanty-Mansyisk region Yugra "Surgut regional clinical hospital"	Moscow		125468	Russia
4422	The Independent Multidisciplinary Committee on Ethical Review of Clinical Trials	Krasnodar Branch of The S.N. Fyodorov FSBI "Eye microsurgery complex"	Moscow		125468	Russia
4425	The Independent Multidisciplinary Committee on Ethical Review of Clinical Trials	SBEI HPE "Saratov State Medical University n.a. V. I. Razumovskiy" of the MoH of the RF	Moscow		125468	Russia
4426	State Autonomous Healthcare Institution "Republican Clinical Opthalmologic Hospital of the Ministry of Health of Tatarstan Republic"	SAIH "Republican clinical ophthalmological hospital of MoH of Rebublic of Tatarstan"	Kazan		420012	Russia
4424	Biomedical Ethics Committee of Federal State Budgetary Institution of scientific and technical complex of Microsurgical eye	MBHI City Clinical Hospital #11	Novosibirs k		630071	Russia
4427	Biomedical Ethics Committee of Federal State Budgetary Institution of scientific and technical complex of Microsurgical eye	The S.N.Fyodorov Federal State Institution Eye Microsurgery Complex (Novosibirsk)	Novosibirs k		630071	Russia
8901	Prince Sultan Military Medical City Research Ethics Committee	Prince Sultan Military Medical City	Riyadh		11159	Saudi Arabia
8902	Kingdom of Saudi Arabia - Ministry of National Guard - Health Affairs	King Abdullah International Medical Research Center IRB Office	Riyadh		22490	Saudi Arabia
8900	King Khaled Eye Specialist Hospital HEC/IRB	King Khaled Eyes Specialist Hospital	Riyadh		11462	Saudi Arabia
8903	Kingdom of Saudi Arabia - Ministry of National Guard - Health Affairs	King Abdullah International Medical Research Center IRB Office	Riyadh		22490	Saudi Arabia
6300	Parkway Hospitals Singapore Pte Ltd	PIEC	Singapore		238164	Singapore
6301	National Healthcare Group	NUHS	Singapore		149547	Singapore
6302	SingHealth Centralised Institutional Review Board	Singapore National Eye Centre	Singapore		168753	Singapore
8600	Republic of Slovenia The National Medical Ethics Committee	Univerzitetni Klinicni Center Ljubljana Ocesna Klinika	Ljubljana		SI-1525	Slovenia

8601	Republic of Slovenia The National Medical Ethics Committee	University Medical Centre Maribor	Ljubljana		SI-1525	Slovenia
8602	Ethical Committee General Hospital Celje	General Hospital Celje	Celje		3000	Slovenia
8603	Republic of Slovenia The National Medical Ethics Committee	General Hospital Novo Mesto	Ljubljana		SI-1525	Slovenia
4500	*Nemocnica Ruzinov*	Univerzitna nemocnica Bratislava, Nemocnica Ruzinov	Bratislava		82606	Slovakia
4502	EK-Fakultna nemocnica Trencin	Fakultna nemocnica Trencin	Trencin		91101	Slovakia
4504	EK-Ustredna vojenska nemocnica SNP Ruzomberok	Ustredna vojenska nemocnica SNP Ruzomberok-Fakultna nemocnica	Ruzomberok		034 26	Slovakia
4507	EK-Fakultna nemocnica s poliklinikou F.D. Roosevelta	NsP Banská Bystrica	Banska Bystrica		975 17	Slovakia
4503	EK-Fakultna nemocnica Zilina	Fakultna nemocnica s poliklinikou Zilina	Zilina		012 07	Slovakia
4501	Eticka komisia NsP Trebisov a.s.	Nemocnica s poliklinikou Trebisov a.s.	Trebisov		075 01	Slovakia
4510	EK-UN Bratislava, Nemocnica sv. Cyrila a Metoda	Univerzitna nemocnica Bratislava, Nemocnica sv. Cyrila a Metoda	Bratislava		85107	Slovakia
4512	EK-Nemocnica Poprad a.s.	Nemocnica Poprad a.s.	Poprad		058 45	Slovakia
4513	EK-FNsP Nove Zamky	Fakultna nemocnica s poliklinikou Nove Zamky	Nove Zamky		94002	Slovakia
4514	Oftal s.r.o, Specializovana nemocnica v odbore oftalmologia	Oftal s.r.o.	Zvolen		960 01	Slovakia
4517	Rozhodnutie Etickej komisie Nemocnica Svateho Michala, a.s.,	Nemocnica svateho Michala	Bratislava		811 08	Slovakia
4900	Ankara University Ethics Committee	Hacettepe University Medical Faculty	Ankara		061 00	Turkey
4901	Ankara University Ethics Committee	Ankara University Medical Faculty	Ankara		061 00	Turkey
4902	Ankara University Ethics Committee	Bilim University Florence Nightingale Hospital	Ankara		061 00	Turkey
4904	Ankara University Ethics Committee	Gazi University Hospital	Ankara		061 00	Turkey
4903	Ankara University Ethics Committee	Ankara Ataturk Training and Research Hospital	Ankara		061 00	Turkey
8700	State Institution "The Filatov Institute of Eye Diseases and Tissue Therapy of the National Academy of Medical Sciences of Ukraine"	V.P.Filatov Institute of Eye Diseases and Tissue Therapy AMS	Odessa		65061	Ukraine
8701	Local Ethics Committee at the Oppthalmology Clinic Eye Microsurgery Center	City clinical ophthalmological hospital	Kyiv		3680	Ukraine
8702	Local Ethics Committee at the Communal Institution Dnipropetrovsk Regional Clinical Ophthalmological Hospital	Regional clinical hospital n.a. Mechnikova	Dnipropetrovsk		49005	Ukraine
8703	Local Ethics Committee at the Hospital	LLC “Lugansk Regional Central Eye Hospital”	Lugansk		91055	Ukraine

8704	Local Ethics Committee at the Kharkov Regional Clinical Hospital	Kharkov Regional Clinical Hospital	Kharkiv		61022	Ukraine
9000	Comité de Etica Independiente de la Fundación Dominicana de Infectología	Consultorio Oftalmológico Medicalnet	Santo Domingo	Santo Domingo	N/A	Dominican Republic
9001	Comité de Etica del Centro Cardio-Neuro Oftalmológico y Trasplante CECANOT	Centro Cardio-Neuro Oftalmológico y Trasplante (CECANOT)	Santo Domingo	Santo Domingo	N/A	Dominican Republic
9031	Instituto Conmemorativo Gorgas de Estudios de la Salud	Clínica de Vitreo y Macula Dra. Ana Paz	Panamá	Panamá	N/A	Panamá
9033	Instituto Conmemorativo Gorgas de Estudios de la Salud	Clinica Yee	Panamá	Panamá	N/A	Panamá
9051	Comité de Etica Zugueme	Clínica Oftalmológica Santa Clara	Guatemala	Guatemal a	1015	Guatemala
9061	Research Ethics Committee - Central Directorate for Research and Health Development	Ministry of Health and Population	Cairo	Cairo	11516	Egypt
9062	Research Ethics Committee - Central Directorate for Research and Health Development	Ministry of Health and Population	Cairo	Cairo	11516	Egypt
9064	Research Ethics Committee - Central Directorate for Research and Health Development	Ministry of Health and Population	Cairo	Cairo	11516	Egypt
9065	Research Ethics Committee - Central Directorate for Research and Health Development	Ministry of Health and Population	Cairo	Alexandria	11516	Egypt
9066	Research Ethics Committee - Central Directorate for Research and Health Development	Ministry of Health and Population	Cairo	Cairo	11516	Egypt
9071	Instituto Costarricense de Investigaciones Clínicas (ICIC)	Instituto de Cirugia Ocular	San José	San José	N/A	Costa Rica
9072	Instituto Costarricense de Investigaciones Clínicas (ICIC)	Clínica 20/20	San José	San José	N/A	Costa Rica
9073	Instituto Costarricense de Investigaciones Clínicas (ICIC)	Oftalmocima	San José	San José	N/A	Costa Rica
1500	Centro Nacional de Bioetica	Unidad Oftalmologica de Caracas, C.A. Santa Paula	Caracas		1060	Venezuela
1501	Centro Nacional de Bioetica	Clinica de Especialidades Oftalmologicas (Retina & Vitreo)	Caracas		1060	Venezuela
1502	Centro Nacional de Bioetica	Centro Oftalmologico de Valencia (CEOVAL)	Caracas		1060	Venezuela
1503	Centro Nacional de Bioetica	Instituto Oftalmológico IUMO trinidad	Caracas		1060	Venezuela
