# Supplementary material for: Effectiveness and safety of ranibizumab 0.5 mg in treatment-naïve patients with diabetic macular edema: Results from the real-world global LUMINOUS study
Source: PLoS One. 2020 Jun 3;15(6):e0233595. doi: 10.1371/journal.pone.0233595 (PMC7269267; doi:10.1371/journal.pone.0233595)
Supplement: S1 File — (DOCX) [file pone.0233595.s003.docx]

The LUMINOUS study confirms the effectiveness of ranibizumab for the treatment of DME in real-world clinical practice and the importance of administering an adequate number of injections and a loading dose. No new safety findings were identified with ranibizumab in this population who are vulnerable to a number of co-morbidities.
